# Delivering maternal and neonatal health interventions in conflict settings: a systematic review

**DOI:** 10.1136/bmjgh-2020-003750

**Published:** 2021-02-19

**Authors:** Mariella Munyuzangabo, Michelle F Gaffey, Dina S Khalifa, Daina Als, Anushka Ataullahjan, Mahdis Kamali, Reena P Jain, Sarah Meteke, Amruta Radhakrishnan, Shailja Shah, Fahad J Siddiqui, Zulfiqar A Bhutta

**Affiliations:** 1 Centre for Global Child Health, The Hospital for Sick Children, Toronto, Ontario, Canada; 2 Health Services and Systems Research, Duke-NUS Graduate Medical School, Singapore; 3 Centre of Excellence in Women and Child Health, Aga Khan University, Karachi, Pakistan

**Keywords:** maternal health, child health

## Abstract

**Background:**

While much progress was made throughout the Millennium Development Goals era in reducing maternal and neonatal mortality, both remain unacceptably high, especially in areas affected by humanitarian crises. While valuable guidance on interventions to improve maternal and neonatal health in both non-crisis and crisis settings exists, guidance on how best to deliver these interventions in crisis settings, and especially in conflict settings, is still limited. This systematic review aimed to synthesise the available literature on the delivery on maternal and neonatal health interventions in conflict settings.

**Methods:**

We searched MEDLINE, Embase, CINAHL and PsycINFO databases using terms related to conflict, women and children, and maternal and neonatal health. We searched websites of 10 humanitarian organisations for relevant grey literature. Publications reporting on conflict-affected populations in low-income and middle-income countries and describing a maternal or neonatal health intervention delivered during or within 5 years after the end of a conflict were included. Information on population, intervention, and delivery characteristics were extracted and narratively synthesised. Quantitative data on intervention coverage and effectiveness were tabulated but no meta-analysis was undertaken.

**Results:**

115 publications met our eligibility criteria. Intervention delivery was most frequently reported in the sub-Saharan Africa region, and most publications focused on displaced populations based in camps. Reported maternal interventions targeted antenatal, obstetric and postnatal care; neonatal interventions focused mostly on essential newborn care. Most interventions were delivered in hospitals and clinics, by doctors and nurses, and were mostly delivered through non-governmental organisations or the existing healthcare system. Delivery barriers included insecurity, lack of resources and lack of skilled health staff. Multi-stakeholder collaboration, the introduction of new technology or systems innovations, and staff training were delivery facilitators. Reporting of intervention coverage or effectiveness data was limited.

**Discussion:**

The relevant existing literature focuses mostly on maternal health especially around the antenatal period. There is still limited literature on postnatal care in conflict settings and even less on newborn care. In crisis settings, as much as in non-crisis settings, there is a need to focus on the first day of birth for both maternal and neonatal health. There is also a need to do more research on how best to involve community members in the delivery of maternal and neonatal health interventions.

**PROSPERO registration number:**

CRD42019125221.

**Key questions**

**What is already known?**

- The insecurity, population displacement and health service disruption that are associated with armed conflict may increase both maternal and neonatal mortality.
- While there is guidance available on the essential interventions for mothers and newborns in humanitarian crises, there is still a lack of guidance on how best to deliver these interventions, especially in conflict settings where there are even more barriers to the access of services.

**What are the new findings?**

- Maternal health interventions were mostly delivered by skilled health workers including doctors and nurses and in hospitals and clinics; the majority of reported interventions focused on antenatal care and its different components.
- Although few, the reported newborn interventions focused on essential newborn care such as neonatal resuscitation and kangaroo mother care, and were also mostly delivered by skilled health workers in clinics and hospitals.
- Among the reported barriers to intervention delivery were the lack of safety, population displacement, and the lack of resources and skilled health workers; however, facilitators included the availability of funding, multisectoral collaboration and the availability of training for staff.
- There is still a wide gap in reporting on newborn health, with very few eligible publications reporting on interventions targeting newborns.

**Key questions**

**What do the new findings imply?**

- There is a need to do more evaluations on the use of lower-cadre health workers and community-based sites in the delivery of maternal health interventions.
- It is also important to assess the feasibility and importance of deploying people from the affected communities, especially in out-of-camp settings where there may not be as many services/resources available.
- There is an urgent need to prioritise newborn health interventions. More research is needed on how best to deliver newborn interventions using lower-skilled health workers and at the community level.

## Introduction

Maternal and newborn mortality remain two of the biggest challenges in global health. Although much progress was made throughout the Millennium Development Goals era, about 810 women still die from pregnancy-related or childbirth-related complications every day. This is especially true in low/middle-income countries (LMICs) where 94% of these deaths occur.^1^ Approximately 7000 newborns also die every day,^2^ accounting for 47% of all under-5 child deaths, and with 36% of them occurring during the first 24 hours after birth and 73% within the first week.^3^ Although it has been shown that increasing coverage of antenatal care (ANC), skilled birth attendance and postnatal care for both the mother and the baby can prevent high maternal and neonatal mortality rates,^4^ data from existing surveys still show that many women and newborns are not reached with these essential interventions.^1^

Within humanitarian settings and especially in the context of armed conflict, insecurity coupled with displacement and service disruption produce an instability that may lead to a higher risk of maternal mortality.^5^ While increased maternal mortality may be a direct effect of armed conflict, it has also been shown that maternal mortality may remain elevated even 1–3 years post-conflict, indicating the longer terms effects of conflict on the disruption of essential health services for pregnant women.^5^ Newborn mortality has also been shown to increase in the context of armed conflict,^6^ and is highest in fragile settings and in countries that have recently experienced a humanitarian crisis.^7^

Comprehensive global guidance exists for addressing maternal and neonatal health (MNH) in non-crisis settings, including the interventions and services outlined in the Every Newborn Action Plan launched in 2014.^8^ Guidance is also available to support MNH in humanitarian crises, including the latest version of the Inter-Agency Field Manual on Reproductive Health in Humanitarian Settings and the Newborn Health in Humanitarian Settings–Field Guide, both released in 2018.^9 10^ However, guidance on how best to deliver recommended interventions for pregnant women and newborns in humanitarian settings, and especially in conflict settings, is still very limited. A review on public health interventions that was included in the 2017 Lancet Series on Health in Humanitarian Crises highlighted the need for higher quality studies on MNH that focus on the most effective means of delivering those interventions in crisis contexts.^11 12^

The present review aimed to systematically synthesise the available indexed and grey literature reporting on the delivery of maternal and newborn interventions in conflict settings. We sought to characterise how and by whom such interventions have been and are being delivered to conflict-affected women and newborns and, if possible, to what extent and with what effectiveness.

## Methods

### Literature search

A systematic search of published literature from 1st January 1990 to 31st March 2018 was conducted; records were retrieved in MEDLINE, Embase, CINAHL and PsycINFO using OVID and EBSCO interfaces. We used sets of terms related to three concepts: (a) conflict; (b) women and children; and (c) MNH. Conflict-related terms included war, crisis, refugees and internally displaced persons (IDPs). Population-related terms included: women, children, pregnant, adolescents and newborn. MNH-related terms included: pregnancy, obstetrics, labour complications and neonatal care, among others. The full MEDLINE search syntax is provided in the online supplemental appendix A. Relevant publications from key systematic reviews^11 13–15^ were also hand searched for potentially relevant publications.

10.1136/bmjgh-2020-003750.supp1 Supplementary data

We searched the websites of 10 major humanitarian agencies and organisations that are actively involved in responding to or researchin conflict situations for grey literature on the implementation of health interventions in pregnant and postpartum women or newborns. These websites included: Emergency Nutrition Network, International Committee of the Red Cross, International Rescue Committee, Médecins Sans Frontières, Save the Children, United Nations (UN) Population Fund, UN High Commissioner for Refugees, UNICEF, Women’s Refugee Commission and World Vision. We used broad terms for conflict and health interventions tailored to the search functionality of each website. Documents published from 1 January 2013 to 30 November 2018 were reviewed.

### Eligibility criteria

Our eligibility criteria included publications reporting on populations affected by conflict in LMICs, as classified by the World Bank.^16^ Eligible publications must have described an MNH intervention targeting or including pregnant or postpartum women or neonates, and delivered during or within 5 years of cessation of a conflict. Where needed, we consulted online encyclopaedic sources as well as the UN Office for the Coordination of Humanitarian Affairs website for information on the duration of a specific conflict, to assess whether the time period of intervention delivery reported in a candidate publication was eligible. In order to identify the most informative resources from the large volume of grey literature available, the same inclusion criteria set for indexed literature was applied, with the additional requirement of explicit reporting on the delivery site and personnel for each intervention.

Non-English publications; case reports of a single patient; or publications reporting on military personnel, refugee populations in high-income countries, surgical techniques, or economic or mathematical modelling studies were excluded from our review. We also excluded systematic and other literature reviews, editorials, commentaries, first-person narratives, newspaper and magazine articles, opinion pieces, guidelines and studies where no specific health intervention was described (eg, prevalence studies).

### Data extraction and analysis

All identified indexed records were downloaded into EndNote software^17^ and duplicates were removed. Unique records were subsequently added into Covidence software for screening.^18^ Two reviewers independently screened titles and abstracts for relevance, and any discrepancies were resolved through discussion or by a third reviewer, if necessary. A single reviewer then screened the full texts of potentially relevant publications to determine their eligibility for inclusion in this review. The same approach was applied to the grey literature, with two reviewers screening titles of retrieved publications for relevance and one reviewer assessing the full text to determine eligibility.

We used a customised form on the REDCap platform^19^ to extract relevant quantitative and qualitative information from all included publications. Data and information on key variables including setting, population characteristics, study design, intervention details and delivery characteristics including reported delivery barriers and facilitators, as well as quantitative data on coverage and effectiveness (when available), were extracted. Two reviewers entered the data in duplicate independently and any inconsistencies identified were resolved through a discussion or by a third reviewer.

Descriptive statistics were performed to summarise key characteristics of the intervention, including displacement status of the target population, delivery characteristics, and reported factors impeding or facilitating delivery. We plotted intervention delivery frequencies stratified by population displacement status, and we mapped reported interventions against reported delivery personnel and site, stratified by intervention type. We tabulated coverage and effectiveness estimates that were reported for pregnant or postpartum women or newborns, but meta-analysis was not conducted due to extensive variability in interventions, outcomes, study settings and populations. All descriptive analyses were conducted using Stata V.14.0 statistical software.^20^ Finally, we narratively synthesised reported information on delivery barriers and facilitators by classifying related information into subgroups and then into broader themes.

### Patient and public involvement

Patients and/or the public were not involved in the design, or conduct, or reporting or dissemination plans of this systematic review of the literature.

## Results

### Characteristics of the included literature

**Figure 1:**
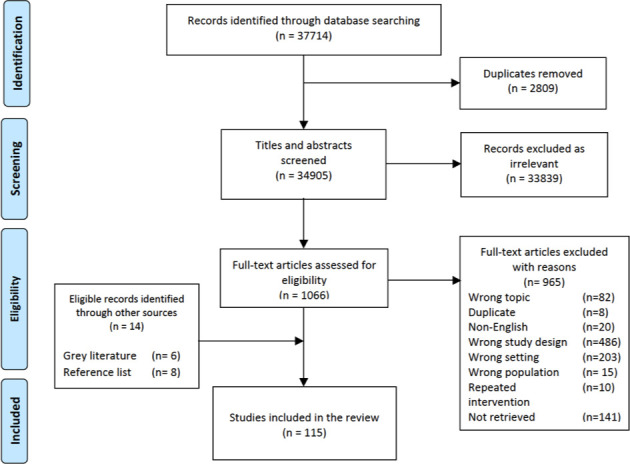
Preferred Reporting Items for Systematic Reviews and Meta-Analyses flow diagram: publication selection process for systematic review on the delivery of maternal and neonatal health interventions in conflict settings.

From 37 714 indexed publications retrieved through our database search, 101 met our eligibility criteria. An additional 14 eligible publications were identified from our grey literature search, and from the reference lists of other reviews. A total of 115 publications were therefore included in this review (figure 1).^21–135^ Of these, only 26 reported on neonatal interventions.^24 26 28 29 33 38 42 44 55 60 63 71 75 97 106 110–113 116 118 120 123 124 133 135^ The characteristics of all included publications are presented in the online supplemental appendix B.

**Figure 2:**
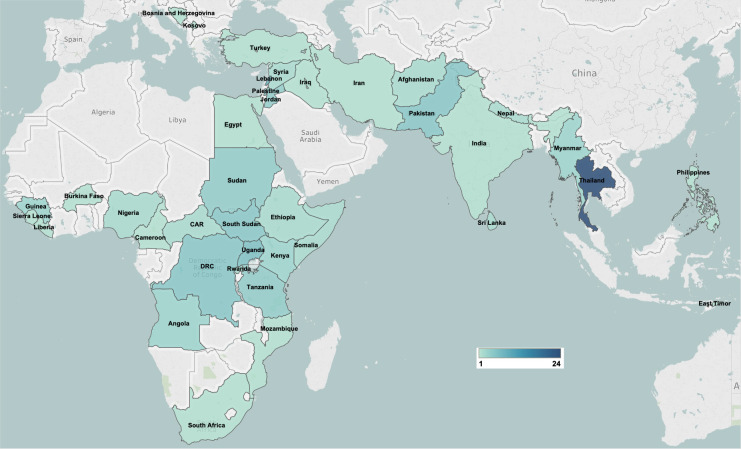
Geographical distribution of included publications.

Of the 115 included publications, MNH intervention delivery was most frequently reported in the sub-Saharan Africa region (43%), followed by the East Asia and Pacific region (27%), mainly in Thailand (figure 2). There were no studies reporting on interventions delivered within the Latin America and Caribbean region. The majority of included publications reported on observational studies (68%), and 17% were non-research reports (table 1).

**Table 1:** Characteristics of included publications and included interventions

**Study and population characteristics (N=115)**	
Geographical region*	**n**
East Asia and Pacific	31
Europe and Central Asia	3
Latin America and the Caribbean	0
Middle East and North Africa	22
South Asia	14
Sub-Saharan Africa	50
Publication type	**n**
Non-research report	19
Mixed methods	11
Observational study	78
Qualitative study	4
Quasi-experimental study	0
Randomised controlled trial	3
Displacement status of beneficiary population*	**n**
Refugees	75
IDPs	36
Non-displaced	20
Returning refugees	2
Host	11
Unreported	12
Setting of displaced populations*†	**n**
Camp	52
Dispersed	16
Mixed	14
Unreported	33
**Intervention delivery characteristics (N=313**)	
Target population type*	**n**
Neonates (<28 days)	32
Pregnant women	253
Postpartum women	51
Implementation platform*	**n**
Existing health system	148
NGO/UN agencies	234
Military based	3
Research based	38

*Publications and interventions can be included in more than one category.

†Only reflects publications that reported on displaced populations (refugees, IDPs or returning refugees).

IDPs, internally displaced persons; NGO, non-governmental organisations; UN, United Nations.

More than half of the included publications reported on interventions delivered to refugees (62%), and about one-third of those delivered to IDPs (27%). There were also a few publications reporting on interventions delivered to non-displaced but conflict-affected populations and to host communities in countries with refugees. Among those publications reporting population displacement settings, intervention delivery to refugees and IDPs was reported most frequently in camp settings (45%). Pregnant women were the most frequently reported intervention target populations, and most reported interventions were delivered by non-governmental organisations (NGOs) (including community-based organisations) or UN agencies, either through the existing healthcare system or in parallel.

### Maternal health interventions

**Figure 3:**
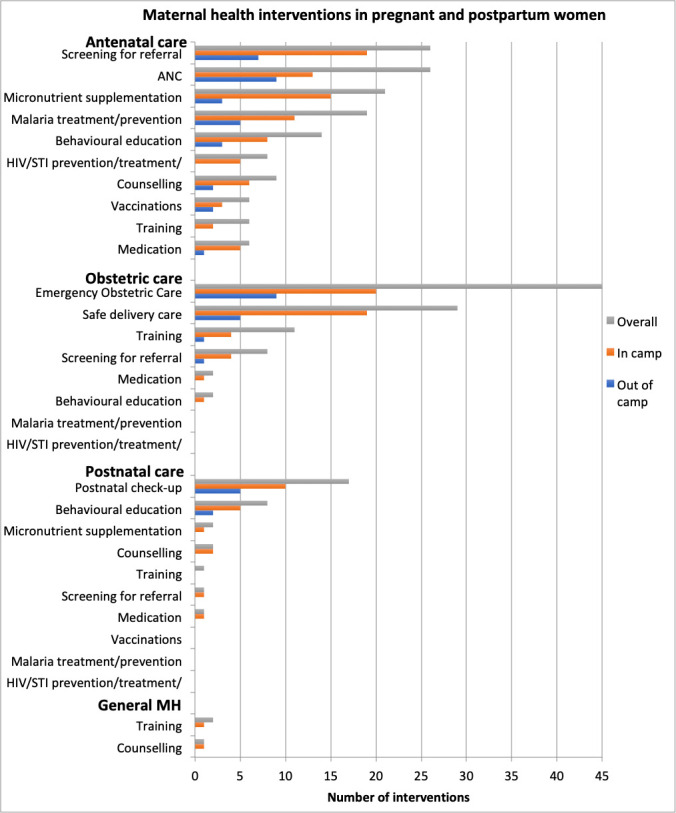
Maternal health interventions in pregnant and postpartum women. ANC, antenatal care; MH, maternal health; STI, sexually transmitted infection.

The reported interventions targeting pregnant or postpartum women covered different components of maternal health such as antenatal, obstetric and postnatal care, as well as general maternal health (figure 3). Within ANC, the most commonly reported interventions were screening interventions for HIV, sexually transmitted infections (STIs), malaria and anaemia; micronutrient supplementation; malaria prevention and treatment; and behavioural education activities. Of the publications that reported on the delivery of ANC, about 35% of them did not specify the components provided. Reported obstetric care interventions were mostly safe delivery care interventions and the provision of basic and comprehensive emergency obstetric care (EmOC), including caesarean sections. Other obstetric care interventions included training of healthcare workers and referral for care. Reported postnatal care interventions included postnatal check-ups within 48 hours of delivery, education activities and micronutrient supplementation among others. Reported general maternal health interventions mainly included training healthcare workers and counselling interventions.

When comparing mothers living in camps with those living outside of camps, more interventions were reported to be delivered to mothers based in camps (figure 3). Some interventions were not reported at all for women living outside of camps, such as training of health workers or interventions targeting the prevention of HIV and STIs during ANC. There were also very few postnatal care interventions and no general maternal health interventions reported to be delivered to out-of-camp mothers.

### Delivery characteristics of maternal health interventions

#### Personnel

**Figure 4:**
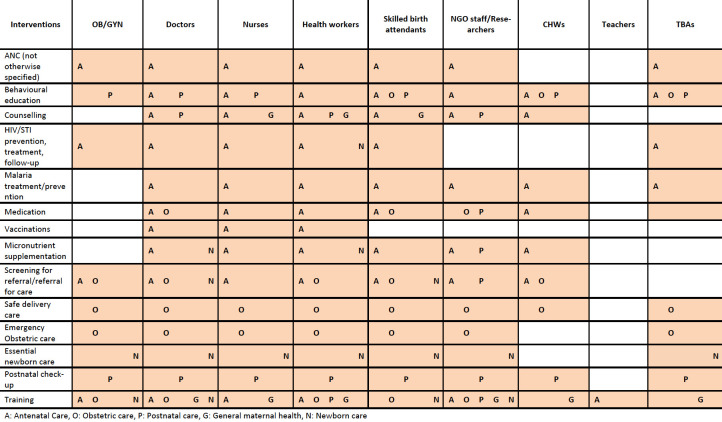
Reported maternal and neonatal health interventions by delivery personnel and intervention type. ANC, antenatal care; CHWs, community health workers; NGO, non-governmental organisation; STI, sexually transmitted infection; TBAs, traditional birth attendants.

Trained health professionals were commonly reported personnel for maternal health interventions. These included doctors, nurses, health workers, skilled birth attendants (SBAs) and OB/GYN specialists (figure 4). NGO staff and researchers were also reported to have delivered most maternal health interventions such as ANC, behavioural education, postnatal care and training. Community health workers (CHWs) were reported to deliver ANC interventions such as malaria treatment and prevention, micronutrient supplementation, behavioural education and counselling among others. Traditional birth attendants (TBAs) were not only involved in delivery care, but were also able to deliver some ANC interventions such as malaria prevention, medication and postnatal check-ups. There were no interventions delivered by community members such as civic leaders or trained civilians and there was only one training intervention where teachers were involved, which was a literacy programme for women.

When we compare in-camp and out-of-camp populations, in-camp mothers were reported to have access to a wider range of personnel compared with out-of-camp populations (data not shown). In-camp interventions were delivered by health workers, nurses, doctors and OB/GYN specialists, while for out-of-camp populations, health workers were the most reported delivery personnel, but with fewer specified professionals reported such as OB/GYN specialists, doctors and nurses. There was also only one reported instance of a TBA being used in an out-of-camp population and very few interventions were reported to be delivered by SBAs or NGO staff. There were few differences in delivery personnel between refugees and IDPs (data not shown), except that while CHWs are mainly used for behavioural education and referral in refugee populations, they were also used for the ANC interventions such as micronutrient supplementation or malaria prevention in IDP populations.

#### Sites

**Figure 5:**
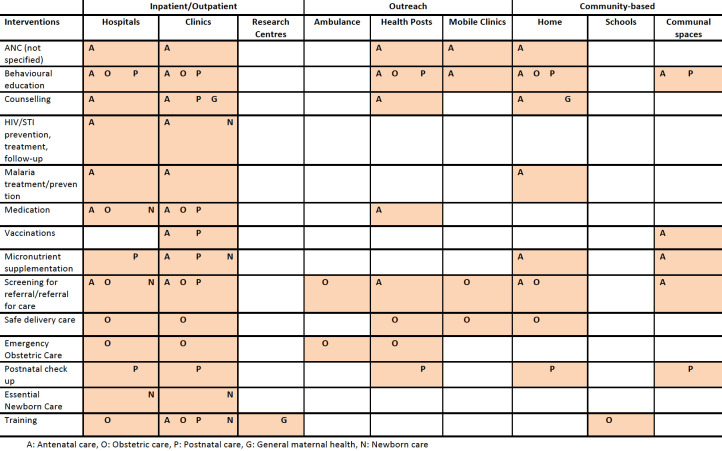
Reported maternal and neonatal health interventions by delivery site and intervention type. ANC, antenatal care; STI, sexually transmitted infection.

Maternal health interventions were mostly delivered using the inpatient and outpatient platforms, with most interventions reported to be delivered within either hospitals or clinics (figure 5). Other common delivery sites included health posts and mobile clinics, mostly for the delivery of ANC interventions. Community-based sites such as homes and communal spaces were also used for ANC and postnatal care interventions. There were few differences between in-camp and out-of-camp populations in terms of delivery sites (data not shown). There were no MNH interventions reported to be delivered in a hospital within out-of-camp populations, and there was also more use of communal spaces for delivery reported within these populations. Interventions targeted towards refugees were delivered more often in clinics and hospitals, with no reports of delivery using outreach sites such as health posts and mobile clinics and very few reporting delivery at the household (data not shown). Interventions targeted towards IDPs used outreach approaches more frequently, with interventions delivered at health posts and through mobile clinics.

### Newborn health interventions

**Figure 6:**
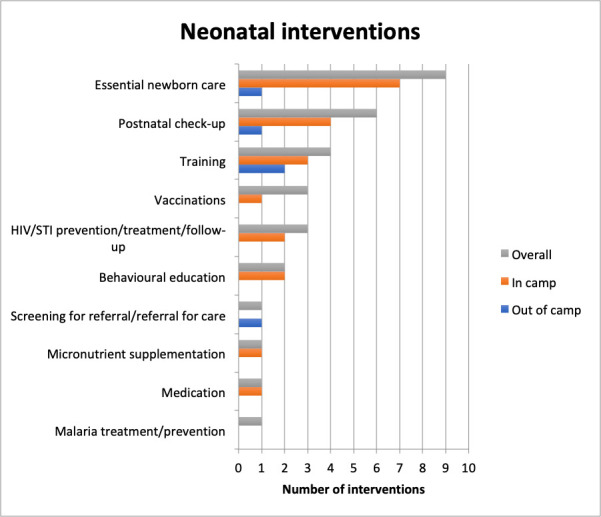
Neonatal health interventions. STI, sexually transmitted infection.

Very few studies reported on newborn care, and only about 10% of all interventions reported were targeting newborns. These interventions were focused mostly on essential newborn care such as neonatal resuscitation, kangaroo mother care and infection prevention. Other common interventions were postnatal check-ups, training of health workers and HIV prevention or follow-up through prevention of mother-to-child transmission (figure 6).

There were more reported interventions on newborns living in camps (69%) compared with those living out of camps (16%). The three most common newborn interventions in camps were essential newborn care, postnatal check-ups and training, while the most common intervention outside of camps was health worker training (figure 6). There were no reported instances of interventions targeting HIV or malaria or delivering vaccinations or micronutrient supplementation among newborns outside of camps.

### Delivery characteristics of newborn health interventions

#### Personnel

Newborn interventions were delivered by SBAs, doctors, nurses, health workers and NGO staff (figure 4). NGO staff were reported to deliver essential newborn care and training interventions. Trained TBAs were also reported to deliver essential newborn care. There were no reports of interventions delivered by CHWs or trained community members. There were no major differences between delivery personnel used for refugee compared with IDP populations. However, there were no reports of OB/GYN specialists being used to deliver newborn interventions among IDP populations. There were also no reports of nurses delivering newborn interventions in refugee populations. There were very few observations of interventions delivery among non-displaced newborns.

#### Sites

All newborn interventions were delivered in hospitals and clinics (figure 5) There were no reported newborn interventions delivered from an outreach or community-based platform. With very limited reporting on newborn interventions delivered outside of camps, it is not possible to compare delivery sites by camp status.

### Reported coverage and effectiveness of MNH interventions

Full details of the retrieved intervention coverage and effectiveness data are presented in the online supplemental appendices C and D. Most estimates were derived from camp-based refugee populations. The majority of reported outcomes in pregnant women assessed coverage of ANC interventions such as the proportion of women accessing four or more ANC visits or the proportion of women receiving different components of ANC such as behavioural education, HIV/STI testing or malaria treatment. Other commonly reported coverage outcomes were on obstetric care such as the rate of caesarean sections and other major obstetric interventions. There were very few outcomes on postnatal care, with only two publications reporting on the proportions of postpartum women attending a postnatal consultation.

The majority of maternal intervention effectiveness estimates reported on the odds of receiving all ANC medical tests or visits, or of delivering at home in women who had received a maternal and child health handbook or maternal education compared with those who had not. There were also a small number of estimates reported regarding the effect of having a CHW in a village on the likelihood of a pregnant women receiving ANC or of having SBA at birth.

The included publications reported very few coverage or effectiveness estimates for newborn interventions. Coverage estimates included those for initiation of breast feeding, kangaroo mother care, antiretroviral (ARV) prophylaxis and healthcare worker training. Effectiveness estimates reported on the rate of exclusive breast feeding after an educational intervention and the rate of death in infants in a special care baby unit after training health workers.

No meta-analysis of intervention coverage or effectiveness data was undertaken given the wide variation in outcome definitions and measures, population characteristics and study designs.

### Barriers to and facilitators of intervention delivery

There were multiple reported barriers to delivering MNH interventions in conflict settings; key barriers and examples are presented in table 2. The most commonly reported barriers were security constraints and displacement. Populations are often not able to access healthcare facilities due to security issues, and in some cases, because facilities have been destroyed. Continuous population movement also limits women’s access to services, and as health workers are likely also affected by the conflict, there may be staff shortages and high turnover. Other reported barriers include lack of infrastructure and resources to provide EmOC services, lack of specialised health workers and stigma towards refugees. A few publications also mentioned a lack of guidelines, such as newborn-specific protocols.

**Table 2:** Reported barriers and facilitators in the implementation of MNH interventions

Barriers	
Security	Being in an insecure environment was often mentioned as a hindrance to the delivery of interventions. Health facilities are destroyed, patients were also unable to access clinics due to security issues. This was especially relevant for services such as emergency obstetric care when women may need to be referred to hospitals.
Lack of resources	Shortages of supplies/resources (medicine, diagnostic tests) during a period of conflict were also noted as barriers and hampered both maternal and newborn care. Materials such as gloves for delivery, obstetric equipment or beds for premature babies were some of the ones noted as missing.^43 111^
Population movement	The continuous population movement limits both delivery and access to health services.
Lack of skilled health workers	The limited training of health workers was a major barrier in the delivery of interventions such as obstetric and newborn care. A few studies mentioned the lack of obstetric specialists as a barrier.^43 104^ A short training although helping to increase knowledge was also noted as not being enough.
Social norms/stigma	This was noted as a barrier for both patients as well as healthcare workers. Refugees may also be stigmatised by their hosts.^63^
Lack of funding	Limited funding was also noted as a barrier as it limited to range of services and materials available.
Limited movement for the women/cost barriers	Conflict reduces means of generating income, especially during displacement. Therefore, the cost of getting health services or of transport might be weighed against other priorities. In some instances where healthcare is not subsidised, cost of care also influenced where women delivered.^49^
Staff affected by conflict	Health services were also limited as staff are also affected by displacement and security concerns. Shortage of staff was a big concern and in some areas was caused by the warring party. In one study, the Taliban were preventing female health providers from working.^106^ Some areas also experienced a high staff turnover, especially in a prolonged conflict.^134^
Limited services	Conflict reduces the range of available services. For maternal health, this was especially dangerous in cases where there is limited EmOC services as needed surgical facilities were not available or there was no training in the management of postpartum complications,^33 64^ emergency referral services were also not always available.
Quality of care	There were some differences noted between the quality of care delivered at hospitals, compared with clinics; hospitals having a higher quality.^111^ ANC and postnatal care were also not always delivered to their full extent.
Lack of guidelines	A few studies mentioned lack of guidelines for STI prevention or lack of newborn-specific clinical and referral protocols as barriers to implementation.^112^
**Facilitators**	
Collaboration	Multisectoral collaboration between international NGOs, the Ministry of Health and existing district health offices/public sector was noted as facilitators. Working with local NGOs was also a facilitator as they are already connected to the community.
Staff training	Training improved the skills of health workers and increased motivation. Continuous supervision/refresher training was encouraged, especially if provided by trained paediatricians or obstetricians.^122^ S-CORT modules are an innovative approach that focuses on training on the clinical services included in the MISP.^122^ One NGO developed a simple and low-cost 38-hour training course to upgrade the skills of TBAs.^113^
Availability of funding/resources	Having adequate funding allowed for more resources.
CHW involvement/outreach workers	Having maternal CHWs from the same community or refugee population was noted as a facilitator in educating women about maternal health.
Use of existing infrastructure	Using the existing infrastructure facilitated the delivery of interventions.^71 110^
Technological/systems innovations	Improving systems such as introducing an ultrasound in outreach settings was noted as a facilitator,^23^ or introducing ANC into family medicine clinics as shown by Homan *et al*.^57^ Using GIS technology with a mobile clinic was shown as being effective in delivering SRH services to IDPs.^114^ Having a free ambulance along with good infrastructure (roads, telephone network) was a facilitator.^91^
Refugee participation	Refugee participation was noted as a facilitator as it provided manpower and community leadership.^90^ Refugee services run by refugees were shown to be feasible if there is sustained funding and technical assistance.^130^
Dedicated staff	Having dedicated healthcare workers was noted multiple times as being a facilitator.^86 106 121^

ANC, antenatal care; CHWs, community health workers; EmOC, emergency obstetric care; GIS, geographic information system; IDPs, internally displaced persons; MISP, Minimum Initial Service Package; MNH, maternal and neonatal health; NGOs, non-governmental organisations; SRH, sexual and reproductive health; STI, sexually transmitted infection; TBAs, traditional birth attendants.

Despite the barriers, there were other factors that were reported to facilitate the delivery of interventions. The presence of skilled and dedicated staff, as well as having a trained doctor/midwife present to provide mentorship for other staff, were noted as facilitators. Another important facilitator reported was the further training of existing healthcare workers. Involving CHWs and training TBAs from the same populations as those being served were also noted as facilitators; these are usually trusted members of the community with whom local women may feel more comfortable. Other reported facilitators include multisectoral collaboration between NGOs and the existing health system, having adequate funding, and refugee participation, among others (table 2).

## Discussion

### Principal findings

The majority of reported maternal health interventions delivered in conflict settings focused on ANC and its different components. Caesarean sections and the availability of EmOC more broadly were also reported relatively commonly, but with little detail on EmOC components. Postnatal care was reported relatively infrequently, also with little detail about what such care was composed of. Maternal health interventions were reported to be delivered mainly by skilled health workers and within hospitals and clinics, but health posts and mobile clinics were also cited, as well as homes and communal spaces at the community level. We did note some differences in delivery personnel and sites between in-camp and out-of-camp mothers, where out-of-camp mothers are mostly attended to by lower cadres of health workers and where communal spaces are used more often.

There were very few reported newborn health interventions, focusing mostly on essential newborn care delivered by trained health workers, or in some cases, by TBAs. Nearly all reported newborn interventions were delivered within hospitals or clinics, with only one (postnatal check-up) delivered in a health post, and none were delivered using a community-based approach. Due to the limited literature, it is difficult to compare the delivery of interventions for neonates born in camps with those born outside of camps.

Conflict hinders the delivery of MNH interventions and common barriers reported include the lack of safety, population displacement, limited resources and services, and a lack of skilled health workforce. Where the workforce is available, there are other barriers such as lack of clear guidelines. However, other factors such as availability of funding, multisectoral collaboration, training and supervision of staff, as well as involving CHWs and refugees, were all reported to have facilitated the delivery of MNH interventions.

Most publications that included quantitative data reported on intervention coverage, and the majority on ANC coverage specifically. There were very few publications that reported on effectiveness of MNH interventions; some studies did compare the rates of caesarean sections or other major obstetric interventions before and after the introduction of an intervention such as the training of health workers. Reported quantitative data on newborn health were mainly coverage estimates of the initiation of ARV prophylaxis in infants exposed to HIV and on initiation of breast feeding. It was not possible to infer differences in intervention coverage or effectiveness by delivery characteristics because of the large variability in outcome measures and populations.

### Evidence gaps

No publications reported on the use of lay community members in the delivery of MNH interventions in conflict settings. It has already been shown that involving community members, including men and community leaders, in the promotion and delivery of maternal health interventions such education/campaigns can be highly effective,^136^ and refugee participation was also noted as a facilitator in one of the studies.

We found very limited literature on newborn care, perhaps reflecting the relative neglect that this area continues to endure in LMICs more generally. However, this may also simply reflect relatively limited reporting of newborn care interventions in the literature to date, rather than actual intervention priorities in the field. While it is encouraging that most reported neonatal interventions were able to be delivered in hospitals and clinics, there may be missed opportunities to use outreach and community-based sites such as health posts or homes, especially when it comes to postnatal care and follow-up for diseases such as HIV. Lower cadre health workers such as CHWs, who seem to be underused when it comes to newborn health, could also be used to deliver these interventions.

There was limited literature on the implementation of the Minimal Initial Service Package (MISP), which is considered key, universal guidance for sexual and reproductive health at the onset of an emergency, including for reducing maternal and neonatal mortality. While a few articles did mention following the MISP guidelines, these did not provide further details on which components. Few publications reported quantitative data, and of those that did, most reported on intervention coverage, mainly ANC coverage. However, even within this one area, there is much variability between publications with respect to how coverage is defined and reported, including with respect to whether such care was provided by skilled health personnel. There is also very limited evidence reported about the effectiveness of these interventions.

Overall, despite the inclusion in this review of many publications reporting on the delivery of MNH interventions, there remains limited information in the literature on how interventions are delivered, and limited consistency with respect to which aspects of delivery are reported and in how much detail. Stronger scientific reporting of intervention delivery in both the indexed and grey literature, including more detailed information on how interventions were implemented, where and by whom, could ultimately inform and improve future programmatic decision-making in the field.

### Study strengths and weaknesses

While we sought to retrieve a comprehensive selection of the grey literature reporting on MNH intervention delivery in conflict settings, the very large volume of potentially relevant reports meant that we had to restrict our search, and we may not have captured the full range of relevant interventions and their delivery characteristics. Moreover, at least some gaps in the existing literature likely reflect reporting gaps rather than actual programmatic gaps, given the many constraints on data collection in conflict settings and on humanitarian health responders’ time and research capacities. Nonetheless, this is the first review, to our knowledge, to systematically capture and synthesise reported information in both the indexed and grey literature on how MNH interventions targeting conflict-affected populations have been and are being delivered in the field.

## Conclusion

While the challenges of research in humanitarian crises make it difficult to determine how well the existing literature on MNH intervention delivery in conflict settings reflects what is actually happening in the field, our results indicate a number of potential implications for research, programming and policy. There is a clear need for more and better reporting on how, where and by whom essential interventions are delivered in conflict settings, and more and better evaluation of the effectiveness of the delivery platforms used. In particular, robust evaluation of the wider use of lower level health workers, including those recruited from affected communities, to deliver a range of MNH interventions outside of camp settings would be especially useful. Further, despite the continued high burden of neonatal mortality in crisis settings, the delivery of newborn health interventions in conflict settings still does not appear to be sufficiently prioritised. This needs to change, urgently.
