# Supplementary material for: Delivering maternal and neonatal health interventions in conflict settings: a systematic review
Source: BMJ Glob Health. 2021 Feb 19;5(Suppl 1):e003750. doi: 10.1136/bmjgh-2020-003750 (PMC7903125; doi:10.1136/bmjgh-2020-003750)
Supplement: Supplementary data [file bmjgh-2020-003750supp001.pdf]

Appendices

Appendix A: MEDLINE Search Strategy

Conflict-related terms

1. disasters/ or emergencies/ or mass casualty incidents/

2. disaster victims/

3. ((disaster or disasters or catastrophe or catastrophes) adj5 (environ* or human or manmade or "man made" or nature or natural or weather)).tw,kf.

4. ("mass casualty" or "mass casualties" or "mass fatalities" or "mass fatality").tw,kf.

5. ((crisis or crises) adj5 (environ* or human or manmade or "man made" or nature or natural or weather)).tw,kf.

6. "warfare and armed conflicts"/ or armed conflicts/ or warfare/ or biological warfare/ or bioterrorism/ or chemical warfare/ or chemical terrorism/ or nuclear warfare/ or psychological warfare/ or war crimes/ or ethnic cleansing/ or genocide/ or holocaust/ or war exposure/ or war-related injuries/

7. afghan campaign 2001-/ or gulf war/ or iraq war, 2003-2011/

8. ("afghan campaign" or "armed conflict" or "armed conflicts" or "gulf war" or "iraq war" or "war time" or "wartime").tw,kf.

9. ((armed or zone or political or civil) adj3 (conflict or conflicts or attack or attacks or war or wars or "no fly")).tw,kf.

10. ("war related injuries" or "war related traumas" or "war related injury" or "war related trauma").tw,kf.

11. ("militant group" or "militant groups" or "militant organization" or "militant organizations" or "militant organisation" or "militant organisations").tw,kf.

12. ("biological terrorism" or bioterrorism or biowarfare or "chemical terrorism" or "ethnic cleansing" or "ethnic cleansings" or "gas poisoning" or genocide or holocaust or holocausts or "nuclear terrorism" or "war exposure" or "war exposures").tw,kf.

17. Starvation/

18. (famine or famines or starvation or starvations).tw,kf.

19. cyclonic storms/ or droughts/ or floods/ or tornadoes/ or tidal waves/

20. avalanches/ or earthquakes/ or landslides/ or tidal waves/ or tsunamis/ or volcanic eruptions/

21. (avalanche or avalanches or cyclone or cyclones or drought or droughts or earthquake or earthquakes or flood or flooded or flooding or floods or hurricane or hurricanes or landslide or landslides or "land slide" or "land slides" or mudslide or mudslides or "mud slide" or "mud slides" or storm or storms or tornado or tornadoes or tsunami or tsunamis or typhoon or typhoons or "volcanic ash" or "volcanic eruption" or "volcanic eruptions" or "volcanic gases").tw,kf.

22. refugees/

23. (evacuee or evacuees or refugee or refugees or squatter or squatters or transients).tw,kf.

24. relief work/ or rescue work/

25. ((rescue or relief or aid) adj (plan or plans or activity or activities or agency or agencies)).tw,kf.

26. ("aid plan" or "aid work" or "relief plan" or "relief work" or "rescue plan" or "rescue work").tw,kf.

27. ((staff or staffs or worker or workers) adj3 (relief or aid)).tw,kf.

28. (humanitarian assistance or humanitarian assistances or relief work or relief works).tw,kf.

29. (humanitarian adj2 (aid or response or relief or crisis or crises or emergency or emergencies or disaster or disasters)).tw,kf.

30. Altruism/

31. (humanitarianism or altruism).tw,kf.

32. ("displaced children" or "displaced families" or "displaced family" or "displaced individuals" or "displaced internally" or "displaced men" or "displaced people" or "displaced peoples" or "displaced person" or "displaced persons" or "displaced population" or "displaced populations" or "displaced women" or "forced displacement" or "forced displacements" or "internal displaced" or "internal displacement" or "internally displaced" or "population displaced" or "population displacement").tw,kf.

33. (((camp or camps) and displac*) or "protected village*").tw,kf.

34. (victim or victims).tw,kf.

35. rubble.tw,kf.

36. or/1-35

Population of interest

37. adolescent/ or young adult/

38. (adolescence or adolescent or adolescents or teen* or youth or youths or "young adult" or "young adults").tw,kf.

39. Pregnant Women/

40. exp pregnancy/

41. (expectant or expectancy or gravid* or pregnant or pregnancies or pregnancy).tw,kf.

42. ("mother to be" or "mothers to be").tw,kf.

43. (prenatal or "pre natal").mp.

44. (perinatal or "peri natal").mp.

45. ((trimester or trimesters) adj3 (first or second or mid or third or final or "1st" or "2nd" or "3rd")).tw,kf.

46. (midtrimester or midtrimesters or "early placental phase" or "early placental phases").tw,kf.

47. exp Delivery, Obstetric/

48. ((labor or labour) adj5 (birth* or breech or childbirth or childbirths or complicat* or difficult or early or easy or induce* or induction or late or obstetric* or onset or pregnan* or present*)).tw,kf.

49. parturients.tw,kf.

50. (birth or births or childbirth or childbirths or parturition or parturitions).tw,kf.

51. ("abdominal deliveries" or "abdominal delivery" or "c-section" or "c-sections" or caesarean or caesareans or cesarean or cesareans or "postcesarean section" or "postcaesarean section").tw,kf.

52. exp Abortion, Induced/

53. (abortion or abortions or embryotomies or embryotomy or "postconception fertility control").tw,kf.

54. ((pregnancy or pregnancies) adj3 terminat*).tw,kf.

55. "sexually active".tw,kf.

56. child/ or child, preschool/ or infant/ or infant, newborn/ or infant, low birth weight/ or infant, small for gestational age/ or infant, very low birth weight/ or infant, extremely low birth weight/ or infant, postmature/ or infant, premature/ or infant, extremely premature/

57. (infan* or newborn* or "new born*" or neonat* or baby* or babies or toddler* or boy or boys or boyfriend or boyhood or girl* or kid or kids or child* or pediatric* or paediatric* or peadiatric* or prematur* or preterm*).mp. or school*.tw.

58. refugees/

59. (refugee or refugees).tw,kf.

60. or/37-59

61. 36 and 60

Domain specific terms – Sexual, Reproductive, Maternal & Neonatal health

62. Gynecology/

63. (gynaecology or gynecology).tw,kf.

64. Reproductive Health/

65. ("reproductive health" or "sexual health").tw,kf.

66. Sex Education/

67. ("family planning education" or "family planning instructor" or "family planning instructors" or "family planning training" or "sex education" or "sex instruction").tw,kf.

68. "minimum initial service package".tw,kf.

69. Maternal Health/

70. Maternal Welfare/

71. ("maternal health" or "maternal welfare" or "maternal child health" or "maternal child welfare").tw,kf.

72. Obstetrics/

73. obstetric*.tw,kf.

74. Pregnancy/

75. (pregnanc* or "child bearing" or childbearing).tw,kf.

76. Prenatal Care/

77. ("prenatal" or "pre natal" or "antenatal" or "ante natal").tw,kf.

78. Perinatal Care/

79. ("perinatal care" or "peri natal care" or "perinatal care" or "peri natal care").tw,kf.

80. Peripartum Period/

81. ("peripartum* period*" or "perinatal* period*" or "peri natal* period*").tw,kf.

82. Parturition/

83. (birth or childbirth* or parturition* or "safe delivery" or "safely delivered").tw,kf.

84. (antepartum or "ante partum" or intrapartum or "intra partum").tw,kf.

85. Stillbirth/

86. (stillbirth or stillbirths or stillborn or stillborns or "still birth" or "still births" or "still born" or "still borns").tw,kf.

87. (emoc or emonc or cemoc or bemoc).tw,kf.

88. Midwifery/

89. ("birth attendant" or "birth attendants" or midwife or midwives or midwifery).tw,kf.

90. Postnatal Care/

91. Postpartum Period/

92. (postnatal or "post natal" or postpartum or "post partum" or puerperium or puerperal).tw,kf.

93. ("neonatal care" or "newborn care" or "new born care" or "helping babies breathe" or "helping babies survive").tw,kf.

94. ("neonatal health" or "newborn health" or "new born health" or "infant health").tw,kf.

95. Abortion, Spontaneous/

96. miscarriage*.tw,kf.

97. abortion, induced/ or abortion, eugenic/ or abortion, legal/ or abortion, therapeutic/

98. Abortion, Septic/

99. (abortion or abortions or aborted or aborting or abortus).tw,kf.

100. Misoprostol/

101. (misoprostol or cityl or cyprostol or cytolog or cytotec or gastotec or gastrul or glefos or gymiso or hemoprostol or isprelor or misel or misodel or misofar or misoone or misopress or misoprostil or misotrol or mispregnol or mysodelle or topogyne).tw,kf.

102. Mifepristone/

103. (corlux or corluxin or korlym or lunarette or mifegest or mifegyne or mifeprex or mifepristone or pictovir or "38486" or "r38486" or "r-38486" or "ru 38 486" or "ru 38486" or "ru 486" or "ru38486" or "ru-38486" or "ru486" or "ru-486" or "vgx 410" or "vgx 410c" or "vgx410" or "vgx410c" or "zk 98296" or "zk98296" or "zk-98296").tw,kf.

104. Birth Intervals/

105. ("birth interval" or "birth intervals" or "birth spacing" or "birth spacings" or "child spacing" or "child spacings" or "family building" or "family planning" or "pregnancy interval" or "pregnancy intervals").tw,kf.

106. "safe motherhood".tw,kf.

107. contraception/ or coitus interruptus/ or contraception, barrier/ or contraception, postcoital/ or natural family planning methods/

108. ("birth regulation" or "birth control*" or "coitus interruptus" or "fertility control" or contracept* or "conception control" or antifertility or anticonception or "fertility control" or "fertilization inhibition" or "inhibition of fertilization" or "fertilisation inhibition" or "inhibition of fertilisation").tw,kf.

109. exp Contraceptive Devices/

110. ("cervical cap" or "cervical caps" or "cervix cap" or "dalkon shield" or "dana deveice" or "i.u.d." or "intra uterine device*" or "intracervical device" or "intrauterine device*" or "intrauterine devices" or "intravaginal pessaries" or "intravaginal pessary" or "iucd" or "iud" or "iuds" or "Lippes loop" or "marguilies spiral" or "margulie coil" or "progestin implant" or "vagina pessaries" or "vagina pessary" or "vaginal diaphragm" or "vaginal diaphragms" or "vaginal pessaries" or "vaginal pessary" or "vaginal ring" or "vaginal shield" or "vaginal rings" or "vaginal shields" or "vaginal sponge" or "vaginal sponges" or condom or condoms).tw,kf.

111. Contraceptives, Postcoital/

112. ("morning after pill" or "postcoital antifertility agent" or "postcoital pill").tw,kf.

113. (ulipristal or uliprisnil or "cdb 2914" or "cdb2914" or "ella (drug)" or "ella one" or "ellaone" or "esmya" or "hrp 2000" or "hrp2000" or "rti 3021 012" or "rti 3021-012" or "rti3021 012" or "rti3021-012" or "ru 44675" or "ru44675" or "va 2914" or "va2914").tw,kf.

114. Sexual Abstinence/

115. (celibacy or "postpartum abstinence" or "sexual abstinence" or virginity).tw,kf.

116. hiv/ or hiv-1/ or hiv-2/

117. Human immunodeficiency virus/

118. ("acquired immune deficiency syndrome virus" or "acquired immunodeficiency syndrome virus" or "aids associated lentivirus" or "aids associated retrovirus" or "aids associated virus" or "aids related virus" or "aids virus" or "aids viruses" or "hiv" or "human immuno deficiency virus" or "human immunodeficiency virus" or "human immunodeficiency viruses").tw,kf.

119. HIV Infections/

120. ("hiv infection" or "hiv infections" or "hiv seropositivit*" or "htlv iii infections" or "htlv iii lav infections" or "htlv-iii infection" or "htlv-iii infections" or "htlv-iii-lav infection" or "htlv-iii-lav infections" or "htlv iii seroconversion" or "htlv iii seropositivit*").tw,kf.

121. Acquired Immunodeficiency Syndrome/

122. ("aids" or "acquired immune deficiency syndrome*" or "acquired immuno deficiency syndrome*" or "acquired immunodeficiency syndrome*").tw,kf.

123. Infectious Disease Transmission, Vertical/pc

124. (116 or 117 or 118 or 119 or 120 or 121 or 122) and 123

125. (pmtct or "prevention of HIV mother to child transmission" or "prevention of mother to child transmission" or "eliminate mother to child transmission" or "prevention of mother to child HIV transmission").tw,kf.

126. Sexually Transmitted Diseases/

127. ("sexually transmitted disease*" or "sexually transmitted infection*" or "venereal disease*" or "venereal infection" or "venereal infections").tw,kf.

128. sexually transmitted diseases, bacterial/ or chancroid/ or chlamydia infections/ or lymphogranuloma venereum/ or gonorrhea/ or syphilis/

129. ("calymmatobacterium granulomatis infection" or "chancroid" or "chancroids" or chlamidiosis or "chlamydia infection" or "chlamydia infections" or "Donovania granulomatis infection" or "donovanosis" or "frei disease" or "gonococcal infection" or "gonococcal infections" or "gonococcosis" or "gonococcus infection" or "gonorrhea" or "gonorrheas" or "gonorrhoea" or "granuloma inguinale" or "granuloma venereum" or "great pox" or "inguinal lymphogranulomatosis" or "Klebsiella granulomatis infection" or "lues" or "lymphogranuloma inguinale" or "lymphogranuloma venereum" or "lymphogranuloma venerum" or "lymphopathia venerea" or "lymphopathia venereum" or "Neisseria gonorrhoeae infection" or "nicholas favre disease" or "nicolas favre disease" or "syphilis" or "syphilitic disorder" or "venereal lymphogranuloma").tw,kf.

130. sexually transmitted diseases, viral/ or herpes genitalis/ or Condylomata Acuminata/

131. ("anal wart" or "anal warts" or "anogenital wart" or "anogenital warts" or "condyla acuminatum" or "condylatum acuminatum" or "condyloma accuminatum" or "condyloma acuminata" or "condylomata acuminata" or "genital herpes" or "genital wart" or "genital warts" or "herpes genitalis" or "herpes progenitalis" or "herpes simplex genitalis" or "herpes simplex virus genital infection" or "penile wart" or "penile warts" or "perianal wart" or "perianal warts" or "venereal wart" or "venereal warts" or "verruca accuminata" or "vulvar condyloma").tw,kf.

132. Scabies/

133. ("sarcoptic mange" or scabies).tw,kf.

134. Phthirus/

135. ("crab lice" or "crab lices" or "crab louse" or "crab louses" or "Pediculus pubis" or "phthirus" or "Phtirus pubis" or "Pthirus pubis" or "pubic lice" or "pubic louse").tw,kf.

136. Intimate Partner Abuse/ or Spouse abuse/ or Domestic Violence/

137. ("partner abuse" or "partner violence" or "wife abuse" or "spouse abuse" or "spousal abuse" or "domestic violence" or "domestic abuse" or "gender based violence" or "sex* based violence").tw,kf.

138. ((abuse* or assault* or violence) adj2 (woman or women)).tw,kf.

139. Sex offenses/ or Human Trafficking/ or Rape/

140. ("coerced intercourse" or "forced prostitution" or "forced sex" or "human trafficking" or "human traffickings" or rape or "sex trafficking" or "sex traffickings" or "sex* abuse*" or "sex* assault*" or "sex*

crime*" or "sex* offense" or "sex* offenses" or "sex* slave*" or "sexual aggression" or "sexual bullying" or "sexual coercion" or "sexual exploitation*" or "sexual harassment" or "sexual trauma" or "sexual violence").tw,kf.

141. ("physical* abuse*" or "physical* assault*" or "physical violence").tw,kf.

142. fistula/ or vaginal fistula/ or rectovaginal fistula/

143. (fistula or fistulas or "genital trauma" or "genital injury" or "vaginal trauma" or "vaginal injury").tw,kf.

144. Resuscitation/ and exp Infant, Newborn/

145. ((resuscitat* or reanimat*) adj3 (neonat* or newborn * or "new born*")).tw,kf.

146. Infant Mortality/ and exp Infant, Newborn/

147. Perinatal Mortality/

148. ("neonat* mortalities" or "neonat* mortality" or "neonatal survival" or "newborn mortalities" or "newborn mortality" or "new born mortalities" or "new born mortality" or "newborn survival" or "new born survival" or "perinatal death rate" or "perinatal mortalities" or "perinatal mortality" or "postneonat* mortality" or "postneonat* mortalities").tw,kf.

149. Hypoxia/ and exp Infant, Newborn/

150. Asphyxia Neonatorum/

151. ("asphyxia neonatorum" or "birth asphyxia" or "neonatal anoxia" or "neonatal asphyxia" or "neonatal hypoxia" or "neonate asphyxia" or "neonatus hypoxia" or "new born asphyxia" or "newborn asphyxia").tw,kf.

152. (care adj3 (cord or cords or umbilical or "funiculus umbilicalis")).tw,kf.

153. umbilical cord/ and prolapse/

154. (prolapse* adj3 (cord or cords or umbilical or "funiculus umbilicalis")).tw,kf.

155. chlorhexidine/

156. ("ay 5312" or "ay5312" or "boston conditioning lotion" or "compound 10040" or "mk 412a" or "mk412a" or "sebidin a" or bidex or chlorhex or chlorhexidin or chlorhexidine or chlorohex or chlorohexidine or chlorohexydine or clohexidine or lisium or nibitane or nolvasan or nolvascin or novalsan or rotersept or sterilon or tubilicid or tubulicid or umbipro).tw,kf.

157. Neonatal Sepsis/

158. ("neonatal early onset sepses" or "neonatal early onset sepsis" or "neonatal late onset sepses" or "neonatal late onset sepsis" or "neonatal sepses" or "neonatal sepsis" or "neonatal septicaemia" or "neonatal septicemia" or "new born sepses" or "new born sepsis" or "new born septicaemia" or "new born septicemia" or "newborn sepses" or "newborn sepsis" or "newborn septicaemia" or "newborn septicemia").tw,kf.

159. Kangaroo-Mother Care Method/

160. ("kangaroo mother care" or "kangaroo mother method" or "skin to skin").tw,kf.

161. exp meningitis/ and exp Infant, Newborn/

162. ((meningitides or meningitis or "meningeal inflammation" or pachymeningitides or pachymeningitis or "perimeningeal infections") adj3 (neonat* or newborn * or "new born*")).tw,kf.

163. Jaundice, Neonatal/

164. ("bronze baby syndrome" or erythroleukoblastosis or ((jaundice or icterus) adj3 (neonat* or newborn * or "new born*"))).tw,kf.

165. Fetal Growth Retardation/

166. ("congenital hypotrophy" or "fetal growth disorder" or "fetal growth restriction" or "fetal growth retardation" or "fetus growth disorder" or "fetus growth retardation" or "foetal growth restriction" or "foetal growth retardation" or "growth retardation in utero" or "in utero growth retardation" or "intrauterine growth restriction" or "intrauterine growth retardation" or "iugr" or "prenatal growth retardation" or "retarded intrauterine growth").tw,kf.

167. Eclampsia/ or Pre-eclampsia/

168. ("eclamptic toxaemia" or "eclamptic toxemia" or "eclamptogenic toxaemia" or "eclamptogenic toxemia" or "edema proteinuria hypertension gestosis" or "eph complex" or "eph gestosis" or "eph syndrome" or "eph toxemia" or "eph toxemias" or "gestational toxaemia" or "gestational toxemia" or "gestational toxicosis" or "hep syndrome" or "hypertension edema proteinuria gestosis" or "pre eclampsia" or "pre eclamptic toxaemia" or "pre eclamptic toxemia" or "preeclamptic toxaemia" or "preeclamptic toxemia" or "pregnancy toxaemia" or "pregnancy toxaemias" or "pregnancy toxemia" or "pregnancy toxemias" or "pregnancy toxicosis" or "proteinuria edema hypertension gestosis" or "toxaemia gravidum" or "toxemia gravidum" or "toxemia of pregnancies" or "toxemia of pregnancy" or "toxemic pregnancy" or eclampsia or eclampsias or preeclampsia).tw,kf.

169. dystocia/

170. ("abnormal labor" or "abnormal labour" or "delayed labor" or "delayed labour" or "inertia uteri" or "labor obstruction" or "labour obstruction" or "obstructed labor" or "obstructed labour" or "uterus inertia" or dystocia or dystocias).tw,kf.

171. Breech Presentation/

172. (breech adj2 (present* or position*)).tw,kf.

173. Uterine Hemorrhage/

174. ("vagina* haemorrhage" or "vagina* hemorrhage" or "vaginal bleeding").tw,kf.

175. or/62-115,124-174

176. 61 and 175

177. limit 176 to dc=20170530-20180331

178. ("2017 05 30*" or "2017 05 31*" or "2017 06*" or "2017 07*" or "2017 08*" or "2017 09*" or "2017 10*" or "2017 11*" or "2017 12*" or "2018 01*" or "2018 02*" or "2018 03*").dt.

179. 176 and 178

180. 177 or 179

BMJ Publishing Group Limited (BMJ) disclaims all liability and responsibility arising from any reliance placed on this supplemental material which has been supplied by the author(s)

Appendix B. Characteristics of included publications

Author	Report type	Country	Displacement status	Displacement setting	Age group	Intervention	Delivery platform	Delivery site	Delivery personnel
Abdelrahim et al (2017)	Observational study	Sudan	IDPs, Not displaced	Camp	Pregnant women	HIV/STI screening for referral, STI prevention, treatment and follow-up care	Healthcare system	Clinics, hospitals	Health workers
Abdulsalam et al. (2003)	Observational study	Syria	Refugees	Dispersed	Pregnant women	Screening for referral	NGO/UN Agency	Clinics	Unreported
Aburas et al. (2017)	Observational study	Syria	IDPs	NR	Pregnant women, neonates	Antenatal care, safe delivery care, postnatal check up	NGO/UN Agency	Clinics	Doctors
Abu-Rustum et al (2015)	Observational study	Lebanon	Refugees, hosts	NR	Pregnant women	Screening for referral	NGO/UN Agency	NR	Obstetrician
Adam at al (2015)	Observational study	Sudan	IDPs	Camp	WRA, Pregnant women, Post-natal mothers	Antenatal care, safe delivery care, postnatal check up, interpersonal communication (counselling), tetanus toxoid vaccinations, behavioural education	NGO/UN Agency	Clinics, home	Doctors, nurses, Health workers, midwives, CHWs
Adam et al (2015)	Observational study	Sudan	IDPs	Camp	WRA, Pregnant women, postnatal mothers, neonates	Counselling, antenatal care, emergency obstetric care, transportation for referral for care, postnatal check up, HIV/STI behavioural education	Healthcare system, NGO/UN Agency	Clinics, home	CHWs, doctors, medical assistant, nurses, skilled birth attendants
Augusto et al (2015)	Observational study	Angola	Not displaced	N/A	Pregnant women	HIV treatment, screening for referral	Healthcare system	Clinics	Unreported
Banks et al (2016)	Observational study	Thailand	Refugees	Camp	Pregnant women, neonates	Counselling, Screening for referral(STIs, and other)	NGO/UN Agency, Research	Clinics	Local counsellors
Bannink-Mbazzi et al. (2013)	Observational study	Uganda	IDPs, Not displaced	Camp	Pregnant women, infants (0-18 months), general population	HIV prevention/treatment/follow-up care, HIV/STI screening for referral, behavioural education	Healthcare system, NGO/UN Agency	Clinics, Electronic/print	Health workers
Bell et al (2016)	Mixed methods study	Rwanda	Refugees	Camp	Pregnant women	Antenatal care	Healthcare system	Clinics	Unreported

Author	Report type	Country	Displacement status	Displacement setting	Age group	Intervention	Delivery platform	Delivery site	Delivery personnel
Benage et al (2015)	Observational study	Lebanon	Refugees	Dispersed	Pregnant women	Screening for referral, breastfeeding promotion and education, vaccinations, iron + folic acid supplementation, contraception provision, postnatal check up (not otherwise specified)	Healthcare system, NGO/UN Agency	Clinics, communal spaces	Health workers
Bile et al (2011)	Non-research	Pakistan	IDPs	Camp, Dispersed	Pregnant women	Breastfeeding promotion and education, contraception provision	Healthcare system, NGO/UN Agency	Clinics	CHWs
Bouchghoul et al (2015)	Observational study	Jordan	Refugees	Camp	Pregnant women, neonates	Screening for referral, Safe delivery care , C-sections, contraception provision, breastfeeding promotion and education, postnatal check-up, emergency hospital transfer for referral	NGO/UN Agency	Clinics, Hospitals	Skilled birth attendants, gynecologists
Brooks et al (2017)	Mixed methods study	DRC	IDPs, Not displaced	Camp	Pregnant women	Malaria prevention	Healthcare system, NGO/UN Agency	Clinics	Unreported
Carrara et al (2006)	Observational study	Thailand	IDPs, Refugees, Not displaced , Hosts	Camp, Dispersed	Pregnant women	Malaria treatment, screening for referral	Healthcare system, Research	Clinics	Health workers (malaria workers)
Carrara et al (2011)	Observational study	Thailand	Refugees, Migrants	Camp, Dispersed	Pregnant women	Screening for referral with intent to treat (other than STIs), Safe delivery care	Research	Clinics	Health workers, skilled birth attendants
Carrara et al (2017)	Observational study	Thailand	Refugees	Camp	Pregnant women	Screening for referral, Multiple micronutrients supplementation, pregnancy-specific food ration	Healthcare system, NGO/UN Agency	Clinics	Health workers
Cetorelli et al (2013)	Observational study	Iraq	Unreported	N/A	Neonates	Polio vaccination	Healthcare system, NGO/UN Agency	NR	Unreported
Cossa et al (1994)	Observational study	Mozambique	IDPs	Camp	Pregnant women	STI screening for referral, syphilis treatment, counselling	Healthcare system	Health posts	Doctors,Nurses

Author	Report type	Country	Displacement status	Displacement setting	Age group	Intervention	Delivery platform	Delivery site	Delivery personnel
Culbert et al (2007)	Observational study	DRC	IDPs, Not displaced	NR	Pregnant women	PMTCT services	Healthcare system, NGO/UN Agency	Clinics, hospitals	Doctors,Nurses, trained volunteers, Health workers
Deboutte et al (2013)	Observational study	DRC	IDPs, Not displaced	Dispersed	Pregnant women	Emergency obstetric care (C-sections)	Healthcare system, NGO/UN Agency	Clinics, hospitals	Doctors, OB/GYN
Devine et al. (2017)	Observational study	Thailand	Refugees	Camp	Pregnant women, neonates	Screening and vaccination for HBV	NGO/UN Agency	Clinics	Local counsellors
Dolan et al. (1993)	RCT	Thailand	Refugees	Camp	Pregnant women	Malaria prevention and treatment (ITNs), screening for referral, iron and folic acid supplementation	NGO/UN Agency, Research	Clinics, hospitals	Doctors, Health workers
Duckett et al (1996)	Observational study	Rwanda	Refugees	Camp	Pregnant women	Iron + folic acid supplementation, malaria prevention, food rations	Defence system, NGO/UN Agency	Clinics	NGO staff
Erenel et al (2017)	Observational study	Turkey	Refugees	NR	Pregnant women	Antenatal care (when not otherwise specified), emergency obstetric care (C-sections), multiple micronutrient supplementation	Healthcare system	Hospitals	Unreported
Fabianiet al (2006)	Observational study	Uganda	IDPs	Camp, Dispersed	Pregnant women	Counselling, STI screening for referral	Healthcare system, Research	Clinics, hospitals	Unreported
Fujiya et al (2007)	Observational study	Palestine	Refugees	Camp	Pregnant women	Provision of health insurance, safe delivery	Healthcare system, NGO/UN Agency	Hospitals	Unreported
Furuta et al (2008)	Qualitative study	Sudan	Refugees	Camp	Pregnant women	Screening for referral with intent to treat (other than STIs), Safe delivery care, contraception provision	Healthcare system	Clinics, hospitals, home	Skilled birth attendants
Garnett et al (2012)	Observational study	South Sudan	Refugees, Returning refugees	NR	Pregnant women	Midwifery training	NGO/UN Agency	Schools	Unreported

Author	Report type	Country	Displacement status	Displacement setting	Age group	Intervention	Delivery platform	Delivery site	Delivery personnel
Ghebreyesus et al (1996)	Observational study	Ethiopia	Not displaced	N/A	Pregnant women	Malaria prevention	Healthcare system, NGO/UN Agency	NR	CHWs
Hafeez et al. (2004)	Non-research	Pakistan	Refugees	Camp, Dispersed	Pregnant women	Safe delivery care, training	Healthcare system, NGO/UN Agency	Hospitals	Doctors, Obstetrician
Hammoury et al (2009)	Observational study	Lebanon	Refugees	Camp, Dispersed	Pregnant women	Antenatal care	NGO/UN Agency	Clinics	Doctors, Nurses, Skilled birth attendants
Hartman et al. (2012)	Non-research	South Sudan	Refugees, Not displaced	NR	Infants (0-12 months), Children (13-59 months), Pregnant women	Malaria prevention (distribution of ITNs and IPTp)	Healthcare system, NGO/UN Agency	NR	Unreported
Holt et al (2003)	Observational study	Ethiopia	Refugees	Camp	Pregnant women	STI screening for referral, antibiotics	NGO/UN Agency	Clinics	Health workers
Homan et al. (2010)	Observational study	Kosovo	Unreported	N/A	Pregnant women, Post-natal mothers	Antenatal care, postnatal check up, breastfeeding promotion and education, referral for care, training	Healthcare system, NGO/UN Agency	Clinics	Doctors, Nurses
Hoogenboom et al. (2015)	Observational study	Thailand	Refugees	Camp	Pregnant women	Antenatal care, iron and folic acid supplementation, malaria treatment, emergency obstetric care, postnatal check-up for mothers and babies	NGO/UN Agency	Clinics	Doctors, skilled birth attendants
Huster et al. (2014)	Observational study	Lebanon	Refugees	NR	Pregnant women	Emergency obstetric care (C-sections)	Healthcare system, NGO/UN Agency	Hospitals	Doctors, skilled birth attendants
Hynes et al. (2017)	Observational study	DRC	IDPs, Hosts	NR	Pregnant women, neonates	Emergency obstetric care, essential newborn care	Healthcare system, NGO/UN Agency	Clinics, hospitals	Nurses, Health workers, skilled birth attendants
Ing et al. (2017)	Observational study	Thailand	Refugees	Camp	Post-natal mothers	Postpartum depression screening and referral for care, counselling, medication	Healthcare system, Research	Clinics	Doctors, Researchers, skilled birth attendants, counsellors

Author	Report type	Country	Displacement status	Displacement setting	Age group	Intervention	Delivery platform	Delivery site	Delivery personnel
Jambai et al. (1996)	Non-research	Sierra Leone	IDPs, not displaced	Camp	Pregnant women	Antenatal care	NGO/UN Agency	Clinics	Doctors, Nurses, skilled birth attendants
Kabakian-Khasholian et al. (2013)	Observational study	Lebanon	IDPs	Dispersed	Pregnant women, Post-natal mothers	Antenatal care, postnatal check up	NGO/UN Agency	Clinics	Doctors, skilled birth attendants
Kabakian-Khasholian et al. (2017)	Qualitative study	Lebanon	Refugees	Dispersed	Pregnant women, post-natal mothers, neonates	Antenatal care, safe delivery care, postnatal check up	Healthcare system, NGO/UN Agency	Clinics	Unreported
Kassim et al. (2012)	RCT	Kenya	Refugees	Camp	Pregnant women	Multiple micronutrients supplementation	NGO/UN Agency	Clinics	NGO staff
Khader et al. (2009)	Observational study	Palestine	Refugees	NR	Pregnant women	Iron + folic acid supplementation	NGO/UN Agency	Clinics	NGO staff
Khan et al. (2017)	Randomized control trial	Pakistan	Returning refugees	N/A	Pregnant women	Counselling (Happy Mother, Healthy Child in Ten Steps)	Healthcare system	Home	CHWs
Khawaja et al (2008)	Observational study	Lebanon	Refugees	NR	Pregnant women	Antenatal care	NGO/UN Agency	Clinics	NGO staff
Kibiribiri et al. (2016)	Observational study	South Africa	Refugees	NR	Pregnant women	Antenatal care, iron and folic acid supplementation, tetanus toxoid vaccination	Healthcare system, NGO/UN Agency	Clinics	Nurses, Health workers, skilled birth attendants
Kitabayashi et al. (2017)	Observational study	Palestine	Refugees	Camp	Pregnant women	Antenatal care	Healthcare system, NGO/UN Agency	Clinics, hospitals	Health workers
Krause et al. (2015)	Mixed methods study	Jordan	Refugees	Camp, Dispersed	Pregnant women, neonates	Safe delivery care, emergency obstetric care, newborn resuscitation, training of health workers on the MISP	Healthcare system, Research	Clinics, hospitals	Health workers
Kruk et al. (2010)	Observational study	Liberia	Unreported	N/A	Pregnant women	Emergency obstetric care (basic), HIV testing and counselling	Healthcare system, NGO/UN Agency	Clinics, health posts, hospitals	Doctors, Nurses, Health workers
Lee et al (2008)	Non-research	Philippines	Unreported	N/A	Pregnant women	Antenatal care, postnatal check up, contraception provision, counselling	Healthcare system, NGO/UN Agency	Clinics, health posts, hospitals	Doctors, nurses, midwives, health workers,

Author	Report type	Country	Displacement status	Displacement setting	Age group	Intervention	Delivery platform	Delivery site	Delivery personnel
Luxemburger et al (2003)	Observational study	Thailand	Refugees	Camp	Pregnant women, post-natal mothers, neonates	Antenatal care, iron and folic acid supplementation, malaria treatment, thiamine supplementation	NGO/UN Agency	Clinics	Doctors, Health workers
Madanat et al (2007)	Observational study	Jordan	Refugees	NR	WRA, Pregnant women, Post-natal mothers	Breastfeeding promotion and education	Healthcare system, NGO/UN Agency	Hospitals	Doctors, Nurses
Mayaud et al (2001)	Observational study	Tanzania	Refugees, hosts	Camp	General population, pregnant women	Sexual health education and promotion, condom provision, screening for referral, antibiotics, training	NGO/UN Agency	Clinics	Trained volunteers, peer educators, health workers
Mayhew et al (2008)	Observational study	Afghanistan	Unreported	N/A	Pregnant women, Post-natal mothers	Antenatal care (when not otherwise specified), postnatal care	Healthcare system	Clinics, hospitals	Doctors, nurses, Skilled birth attendants
McGinn et al (2006)	Observational study	Guinea	Refugees	Camp	Pregnant women, Post-natal mothers, women (20-65+ years)	Training	NGO/UN Agency, Research	NR	NGO staff, teachers
McGready et al (2001)	Observational study	Thailand	Refugees	Camp	Pregnant women, Post-natal mothers	postnatal check up, thiamine supplementation	NGO/UN Agency, Research	Clinics	NGO staff
McGready et al. (2010)	Observational study	Thailand	Refugees	Camp	Pregnant women	Antibiotics for maternal infections	NGO/UN Agency	Hospitals	Doctors, skilled birth attendants
McGready et al. (2012)	Observational study	Thailand	Refugees	Camp	Pregnant women	HAART provision, malaria screening and treatment, thiamine and iron and folic acid supplementation	Research	Clinics	Doctors, nurses, skilled birth attendants, health workers
McGready et al. (2015)	Observational study	Thailand	Refugees	Camp	Pregnant women	Syphilis treatment, multiple micronutrient supplementation, tetanus toxoid vaccination, ART provision	NGO/UN Agency	Clinics	Laboratory technicians, skilled birth attendants
McGregor et al. (2017)	Observational study	Thailand	Refugees	Camp	Pregnant women	Screening for referral with intent to treat	Healthcare system, NGO/UN Agency	Clinics, hospitals	Sonographers

Author	Report type	Country	Displacement status	Displacement setting	Age group	Intervention	Delivery platform	Delivery site	Delivery personnel
McPherson et al. (2006)	Observational study	Nepal	Not displaced	N/A	WRA, Pregnant women, Post-natal mothers	Behavioural education (birth preparedness package), training	Healthcare system, NGO/UN Agency	Clinics, Health posts, Home, Hospitals	CHWs, skilled birth attendants, trained TBAs
Mercer et al (2006)	Observational study	India	Refugees	Dispersed	Pregnant women	The routine use of a partogram and the active management of the third stage of labour with intramuscular 'Syntrometrine' (5U Syntocinon and 0.5 mg Ergometrine) as the anterior shoulder is delivered	Healthcare system, NGO/UN Agency	Hospitals	Doctors, Nurses, Skilled birth attendants
Miller et al. (1995)	Non-research	Pakistan	Refugees	Camp	Pregnant women, Post-natal mothers	Antenatal care, breastfeeding promotion, clean delivery kits, safe delivery, postnatal check up, training	NGO/UN Agency	Home	NGO staff, skilled and TBAs
Mohammadi et al. (2017)	Observational study	Iran	Refugees	Dispersed	Pregnant women	Emergency obstetric care (C-sections)	Healthcare system	Hospitals	Unreported
Morrison et al. (2000)	Mixed methods study	Thailand	Refugees	Camp	Pregnant women, post natal mothers, women (15-65+ years)	Antenatal care, safe delivery, contraception provision, STI screening for referral, training	Healthcare system, NGO/UN Agency	Clinics, hospitals	Doctors, midwives, NGO staff, TBAs
Msuya et al. (1996)	Non-research	Tanzania	Refugees	Camp	Pregnant women	STI screening for referral	NGO/UN Agency	Clinics, electronic/print, communal spaces	NGO staff, nurses, skilled birth attendants, health behaviour promoters, peer educators
Mucunguzi et al. (2014)	Observational study	Uganda	Not displaced	N/A	Pregnant women	Emergency obstetric care, transportation for emergency referral	Healthcare system	Hospitals, ambulance	Emergency response team
Muhammad et al. (2016)	Observational study	Nigeria	Unreported	N/A	Pregnant women	Malaria prevention (IPTp, ITNs)	Healthcare system	Clinics	Unreported
Mullany et al (2008)	Non-research	Burma (Myanmar)	IDPs	NR	Pregnant women, Post-natal mothers	Training	Healthcare system, NGO/UN Agency	Research centres	CHWs, health workers and TBAs
Mullany et al. (2010)	Observational study	Burma (Myanmar)	IDPs	Dispersed	Pregnant women	Training on safe delivery and emergency obstetric care	NGO/UN Agency	Clinics	Doctors, Health workers

Author	Report type	Country	Displacement status	Displacement setting	Age group	Intervention	Delivery platform	Delivery site	Delivery personnel
Mullany ey al (2008)	Observational study	Burma (Myanmar)	IDPs	Dispersed	WRA, Pregnant women	Training, malaria treatment, screening for referral, iron + folic acid supplementation	NGO/UN Agency	Clinics, home	Trained surveyors, TBAs
Muller et al. (2015)	Observational study	DRC	Not displaced	N/A	Pregnant women	Training, screening for referral	Healthcare system	Hospitals	Doctors
Musmar (2012)	Observational study	Palestine	IDPs	Camp	Neonates, Post-natal mothers	Breastfeeding promotion and education	NGO/UN Agency	Clinics	Nurses
Nosten et al. (1991)	Observational study	Thailand	Refugees	Camp	WRA, Pregnant women, Post-natal mothers	Malaria treatment, iron and folic acid supplementation, screening for referral, safe delivery, behavioural education	NGO/UN Agency	Clinics	Health workers, TBAs
Nosten et al. (1999)	Observational study	Thailand	Refugees	Camp	Pregnant women	Antenatal care, malaria treatment, iron and folic acid supplementation	Research	Clinics	Health workers
Obol et al. (2013)	Observational study	Uganda	IDPs	Camp	Pregnant women	Malaria prevention (ITN distribution)	NGO/UN Agency	Clinics	NGO staff
Odero et al(2001)	Observational study	Kenya	Refugees, hosts	Camp	Pregnant women	Emergency obstetric care (C-sections, laparotomy, hysterectomy, craniotomy, embryotomy and internal version)	Healthcare system, NGO/UN Agency	Hospitals	Nurses, General medical officers
Orach et al (2004)	Observational study	Uganda	Refugees, hosts	Camp	Pregnant women	Major obstetric interventions	Healthcare system, NGO/UN Agency	Hospitals	Unreported
Parr et al. (2014)	Mixed methods study	Thailand	Refugees	Camp	Pregnant women	Emergency obstetric care (management of foetal nuchal cord ligation)	Research	Clinics	Doctors, skilled birth attendants
Pearson et al (2005)	Mixed methods study	Kenya	Refugees	Camp	Pregnant women	Emergency obstetric care	Healthcare system, NGO/UN Agency	Clinics, hospitals	Doctors, Nurses, Skilled birth attendants
Plewes et al (2008)	Mixed methods study	Thailand	Refugees	Camp	Pregnant women	Contraception provision, HIV treatment and counselling	Healthcare system, NGO/UN Agency	Clinics	Counsellors

Author	Report type	Country	Displacement status	Displacement setting	Age group	Intervention	Delivery platform	Delivery site	Delivery personnel
Purdin et al. (2009)	Observational study	Pakistan	Refugees, hosts	Camp	Pregnant women, post-natal mothers, neonates	Antenatal care, referral for care, emergency obstetric care, postnatal check-up, training	NGO/UN Agency	Clinics, hospitals	Doctors, nurses, Health workers, skilled birth attendants and TBAs, NGO staff
Rijken et al. (2009)	Observational study	Thailand	Refugees	Camp	Pregnant women	Training, screening for referral (Ultrasound imaging for gestational age assessment during ANC visit)	Healthcare system, NGO/UN Agency	Clinics	Doctor, Health workers, CHWs
Ruckstuhl et al (2017)	Observational study	Central African Republic	IDPs, Not displaced	Dispersed	Pregnant women	Malaria prevention and treatment, folic acid supplementation, clean delivery kits, deworming	Healthcare system, NGO/UN Agency	Clinics	CHWs
Rull et al. (2018)	Non-research	South Sudan	IDPs	Camp, Dispersed	Pregnant women	Antenatal care	NGO/UN Agency	Clinics	Unreported
Rutta et al. (2008)	Observational study	Tanzania	Refugees	Camp	Pregnant women, post-natal mothers, neonates, infants (1-18 months)	HIV counselling and testing, HIV prevention, treatment and follow up care (home-based care, nutritional support, infant-feeding counselling), behavioural education, training	Healthcare system, NGO/UN Agency	Clinics, hospitals	CHWs, Doctors, Nurses, skilled birth attendants, Health workers, counsellors
Sami et al. (2017)	Observational study	South Sudan	IDPs, Refugees	Camp	Neonates	Newborn care (neonatal resuscitation, kangaroo mother care, early initiation of breastfeeding, infection prevention), postnatal check up, safe delivery care, screening for referral (partograph use)	NGO/UN Agency	Clinics, hospitals	Skilled and TBAs
Sami et al. (2017)	Mixed methods study	South Sudan	IDPs, Refugees	Camp, Dispersed	Neonates	Training on essential newborn care	Healthcare system, NGO/UN Agency	Clinics	Doctors, NGO staff, skilled birth attendants
Schaider et al. (1999)	Observational study	Angola	Unreported	N/A	Pregnant women, post-natal mothers, neonates	Training of traditional birth attendants	NGO/UN Agency	NR	NGO staff, Health workers
Shaikh et al. (2008)	Non-research	Somalia	IDPs	Camp	Pregnant women	Breastfeeding promotion and education, safe delivery care	NGO/UN Agency	Mobile clinics	Nurses, skilled birth attendants

Author	Report type	Country	Displacement status	Displacement setting	Age group	Intervention	Delivery platform	Delivery site	Delivery personnel
Shrimpton et al. (2009)	Observational study	Nepal	Refugees	Camp	Pregnant women	Multiple micronutrients supplementation, supplementary food ration provision	NGO/UN Agency	Clinics	Unreported
Simetka et al. (2002)	Observational study	Sri Lanka	IDPs	Dispersed	Pregnant women, neonates	Antenatal care (when not otherwise specified), tetanus toxoid vaccination, malaria prevention and treatment, safe delivery care, emergency obstetric care, referral for care	Healthcare system	Clinics, hospitals	Doctors, nurses, skilled birth attendants, OB/GYN, an anaesthetist, a surgeon and a paediatrician.
Sivaganesh et al. (2009)	Observational study	Sri Lanka	IDPs	Dispersed	Pregnant women	Antenatal care (when not otherwise specified)	Healthcare system	Clinics, hospitals, home	Health workers, skilled birth attendants
Somigliana et al. 2011	Observational study	Uganda	Unreported	N/A	Pregnant women, neonates	Antenatal care, ART provision, safe delivery care, emergency obstetric care, newborn care, referral system for obstetric emergencies	NGO/UN Agency	Clinics, Hospitals, ambulance	Health workers, gynaecologists, TBAs
Stein et al. (2008)	Observational study	Tanzania	Refugees	NR	Pregnant women	Emergency obstetric care	Healthcare system	Hospitals	Unreported
Tatah et al. (2016)	Observational study	Cameroon	Refugees, hosts	NR	Pregnant women, neonates	Antenatal care, safe delivery care, emergency obstetric care, counselling, vaccinations for babies	Healthcare system	Clinics	Health workers
Tome et al. (1998)	Non-research	Angola	Unreported	N/A	Pregnant women	Emergency obstetric care (management of pre-eclampsia and eclampsia, antihypertensive medication), safe delivery care, referral for care, training	Healthcare system	Hospitals	Doctors, skilled birth attendants
Tran et al. (2017)	Qualitative study	Burkina Faso, South Sudan	Refugees	Camp	Pregnant women	Emergency obstetric care (manual vacuum aspiration)	Healthcare system, NGO/UN Agency	NR	Health workers
Turner et al. (2013, BMC Inf Dis)	Observational study	Thailand	Refugees	Camp	Pregnant women, neonates	Safe delivery care, antibiotic treatment	Research	Clinics, hospitals	Paediatrician, doctor, nurses, medics, skilled birth attendants

Author	Report type	Country	Displacement status	Displacement setting	Age group	Intervention	Delivery platform	Delivery site	Delivery personnel
Turner et al. (2013,PLoS ONE)	Observational study	Thailand	Refugees	Camp	Neonates	Neonatal resuscitation, phototherapy for jaundice treatment	Research	Clinics, hospitals	Paediatrician, doctor, nurses, medics, skilled birth attendants
Van Damme et al. (1998)	Observational study	Guinea	Refugees, hosts	Camp, Dispersed	Pregnant women	Emergency obstetric care (C-sections), referral for care	Healthcare system, NGO/UN Agency	Hospitals, ambulance	Doctors, Health workers
Viswanathan et al. (2012)	Observational study	Afghanistan	Not displaced	N/A	Adolescents, Women (20-49 years), Pregnant women, Post-natal mothers	Training of CHWs	Healthcare system	Clinics	Health workers
Von Roenne et al. (2010)	Non-research	Guinea	Refugees, hosts	Camp, Dispersed	Pregnant women, Post-natal mothers	Antenatal care, safe delivery care, postnatal check up,	Healthcare system, NGO/UN Agency	Clinics, Hospitals, communal spaces, electronic/print	Nurses, skilled birth attendants, CHWs, drama groups
Wayte et al. (2008)	Mixed methods study	East Timor	IDPs	Camp, Dispersed	Pregnant women	Antenatal care, breastfeeding promotion and education, maternity waiting camps	Healthcare system, NGO/UN Agency	Clinics, hospitals, mobile clinics	Doctors, skilled birth attendants, obstetricians, health workers, NGO staff
West et al. (2016)	Qualitative study	Jordan	Refugees	Camp	Pregnant women, Post-natal mothers	Antenatal care, safe delivery care, postnatal check up	NGO/UN Agency	Clinics	Unreported
White et al. (2012)	Mixed methods study	Thailand	Refugees	Camp	Pregnant women, post-natal mothers, neonates	Multiple micronutrients supplementation, tetanus toxoid vaccination, safe delivery care, breastfeeding promotion and education, newborn care, postnatal care	Research	Clinics, hospitals	Health workers, skilled birth attendants
White et al. (2016)	Mixed methods study	Thailand	Refugees	Camp	Pregnant women	Training	NGO/UN Agency	Clinics	Doctors, skilled birth attendants

Author	Report type	Country	Displacement status	Displacement setting	Age group	Intervention	Delivery platform	Delivery site	Delivery personnel
UNFPA (2016)	Non-research	Nigeria, Pakistan, Burma (Myanmar), Somalia	IDPs, Hosts	Camp, Dispersed	Adolescents, Women >20 years, Pregnant women, Post-natal mothers, General population	MISP, Behavioural education, women friendly health spaces, training, Antenatal care: screening, urine analysis, haemoglobin, referrals) HIV/STIs screening for referral, referral for care for obstetric fistulas, safe delivery care, basic EMOC	NGO/UN Agency	Clinics, Mobile Clinics, Health posts, home, electronic/print	Health workers, doctors, skilled birth attendants, CHWs, NGO
UNHCR (2015)	Non-research	Egypt, Jordan	Refugees	Camp, Dispersed	WRA, Pregnant women	Cash transfer, family planning counselling, behavioural education, referral for care	NGO/UN Agency	Clinics, Hospitals	Health workers, doctors, CHWs
Waddah (2016)	Non-research	Palestine	IDPs, Refugees	Dispersed	Post-natal mothers	Breastfeeding promotion and education, postnatal check up	NGO/UN Agency	Family centers, home, electronic (mobile communication)	CHWs, NGO staff
UNICEF (2016)	Non-research	Central African Republic	IDPs	Camp, Dispersed	Pregnant women	HIV Counselling	NGO/UN Agency	NR	NGO staff
Desie (2017)	Non-research	Somalia	IDPs, Not displaced	Camp, Dispersed	Pregnant women, post-natal mothers, neonates	Breastfeeding promotion and education	NGO/UN Agency	Mobile clinics	Health workers, paramedics

Munyuzangabo M, *et al. BMJ Global Health* 2021; 5:e003750. doi: 10.1136/bmjgh-2020-003750

Appendix C: Reported coverage and effectiveness of maternal health interventions

Author (year)	Country	Target population	Intervention	Report type	Outcome	Description of outcome	Effect measure	Sample size	Estimate (95% CI)	Displace ment status	Setting	Personnel	Site of delivery
**Antenatal care**													
Benage et al. 2015	Lebanon	Pregnant women	Antenatal care (not specified)	Observational study	Coverage	Proportion of Syrian refugee pregnant women who presented at the clinics and accessed the requisite 4 or more antenatal care visits	%	420	15.7 (12.2, 19.18)	Refugees	Dispersed	Health workers	Clinics, communal spaces
Benage et al. 2015	Lebanon	Pregnant women	Antenatal care (not specified)	Observational study	Coverage	Proportion of Syrian refugee pregnant women who presented at the clinics and accessed at least one antenatal care visit	%	420	82.8	Refugees	Dispersed	Health workers	Clinics, communal spaces
Erenel et al. 2017	Turkey	Pregnant women	Antenatal care (not specified)	Observational study	Coverage	Proportion of Syrian refugee pregnant patients who accessed more than 2 antenatal care visits out of the cohort that delivered at the research hospital between January 2013 and January 2016	%	300	17.3 (13.02, 21.58)	Refugees	Not reported	Unreported	Hospitals
Erenel et al. 2017	Turkey	Pregnant women	Antenatal care (not specified)	Observational study	Coverage	Proportion of Turkish pregnant patients (controls) who accessed more than 2 antenatal care visits out of the cohort that delivered at the research hospital between January 2013 and January 2016	%	300	76.3 (71.49, 81.11)	Refugees	Not reported	Unreported	Hospitals
Purdin et al. 2009	Pakistan	Pregnant women	Antenatal care (not specified)	Observational study	Coverage	Proportion of pregnant Afghan refugee women who received complete prenatal care coverage (defined as 3 or more visits) in 2000 as part of the IRC primary health care program	%	0	49	Hosts, Refugees	Camp	Health workers	Clinics
Purdin et al. 2009	Pakistan	Pregnant women	Antenatal care (not specified)	Observational study	Coverage	Proportion of pregnant Afghan refugee women who received complete prenatal care coverage (defined as 3 or more visits) in 2006 as part of the IRC primary health care program	%	0	90	Hosts, Refugees	Camp	Health workers	Clinics

Author (year)	Country	Target population	Intervention	Report type	Outcome	Description of outcome	Effect measure	Sample size	Estimate (95% CI)	Displace ment status	Setting	Personnel	Site of delivery
Simetka et al. 2002	Sri Lanka	Pregnant women	Antenatal care (not specified)	Observational study	Coverage	Proportion of women who had attended at least one antenatal clinic out of all women at who delivered at Mallavi hospital during November 2000-April 2001	%	685	94 (92.22, 95.78)	IDPs	Dispersed	Doctors, nurses, OB/GYN specialists, skilled birth attendants, volunteer attendants	Hospitals
Bouchghoul et al. 2015	Jordan	Pregnant women	Antenatal care (not specified)	Observational study	Coverage	Monthly antenatal consultations in pregnant Syrian refugee women who came to the GSF unit for delivery in February 2013	Rate	371	111	Refugees	Camp	Skilled birth attendants, OB/GYN specialists	Clinics
Carrara et al. 2017	Thailand	Pregnant women	Antenatal care (not specified)	Observational study	Coverage	Proportion of women presenting to an ANC in the first trimester	%	0	76	Refugees	Camp	Health workers	Clinics
Purdin et al. 2009	Pakistan	Pregnant women	Behavioural education	Observational study	Coverage	Proportion of Afghan refugee births attended by skilled staff in an IRC-run EmOC facility in Hangu district 1996	%	0	4.8	Hosts, Refugees	Camp	Health workers	Clinics
Purdin et al. 2009	Pakistan	Pregnant women	Behavioural education	Observational study	Coverage	Proportion of Afghan refugee births attended by skilled staff in an IRC-run EmOC facility in Hangu district in 2007	%	0	67.2	Hosts, Refugees	Camp	Health workers	Clinics
Kitabayashi et al. 2017	Palestine	Pregnant women	Behavioural education	Observational study	Coverage	Proportion of women who were MCH handbook owners out of all women who had live births within the past 12 months nationally	%	2065	60 (57.89, 62.11)	Refugees, not displaced	Camp	Health workers	Clinics, hospitals
Kitabayashi et al. 2017	Palestine	Pregnant women	Behavioural education	Observational study	Effectiveness	Adjusted odds ratio of receiving all 3 medical tests (blood pressure, blood and urine analyses) in ANC in pregnant women who were MCH handbook holders compared to non-holders out of all women who had live births within the past 12 months nationally	Odds ratio	2065	1.7 (1.37, 2.16)	Refugees, not displaced	Camp	Health workers	Clinics, hospitals
Kitabayashi et al. 2017	Palestine	Pregnant women	Behavioural education	Observational study	Effectiveness	Adjusted odds ratio of receiving health information on 5 or more topics in ANC in pregnant women who were MCH handbook holders compared to non-holders out of all women who had live births within the past 12 months nationally.	Odds ratio	2065	1.6 (1.37, 1.95)	Refugees, not displaced	Camp	Health workers	Clinics, hospitals

Author (year)	Country	Target population	Intervention	Report type	Outcome	Description of outcome	Effect measure	Sample size	Estimate (95% CI)	Displacement status	Setting	Personnel	Site of delivery
Augusto et al. 2015	Angola	Pregnant women	HIV prevention, treatment and follow-up care	Observational study	Coverage	Proportion pregnant women testing positive for HIV at PMTCT sites who received ARV prophylaxis nationally in 2005	%	608	71 (67.39, 74.61)	Not displaced	N/A	Unreported	Clinics
Augusto et al. 2015	Angola	Pregnant women	HIV prevention, treatment and follow-up care	Observational study	Coverage	Proportion pregnant women testing positive for HIV at PMTCT sites who received ARV prophylaxis nationally in 2012	%	5805	46 (44.72, 47.72)	Not displaced	N/A	Unreported	Clinics
Bannink-Mbazzi et al. 2013	Uganda	Pregnant women	HIV prevention, treatment and follow-up care	Observational study	Coverage	Proportion of pregnant women who were started on ARV prophylaxis for PMTCT out of the women who had tested HIV-positive at MOH/AVSI health facilities between 2002 and 2011	%	8283	69.4 (68.41, 70.39)	IDPs, not displaced	Camp	Unreported	Clinics
Bannink-Mbazzi et al. 2013	Uganda	Pregnant women	HIV prevention, treatment and follow-up care	Observational study	Coverage	Proportion of pregnant women who were started on triple ART out of the women who had tested HIV-positive at MOH/AVSI health facilities between 2002 and 2011	%	8283	9.6 (8.97, 10.23)	IDPs, not displaced	Camp	Unreported	Clinics
Dolan et al. 1993	Thailand	Pregnant women	Malaria treatment/prevention	RCT	Effectiveness	Relative risk of at least having one attack of falciparum in pregnant women who presented for an ANC visit and received a study impregnated bednet compared to those who received a non-impregnated bednet in Shoklo	Risk ratio	36	2.6 (1.22, 2.72)	Refugees	Camp	Doctors, health workers	Clinics, hospitals
Dolan et al. 1993	Thailand	Pregnant women	Malaria treatment/prevention	RCT	Effectiveness	Relative risk of at least having one attack of falciparum in pregnant women who presented for an ANC visit and who already had a family size non-impregnated bednet compared to those who received a study non-impregnated bednet in Shoklo	Risk ratio	19	4.8 (1.36, 17.27)	Refugees	Camp	Doctors, health workers	Clinics, hospitals
Dolan et al. 1993	Thailand	Pregnant women	Malaria treatment/prevention	RCT	Effectiveness	Relative risk of at least having one attack of falciparum in pregnant women who presented for an ANC visit and received a study impregnated bednet compared to those who received a non-impregnated bednet in Bono and Maesalit	Risk ratio	67	0.9 (0.47, 1.76)	Refugees	Camp	Doctors, health workers	Clinics, hospitals

Author (year)	Country	Target population	Intervention	Report type	Outcome	Description of outcome	Effect measure	Sample size	Estimate (95% CI)	Displacement status	Setting	Personnel	Site of delivery
Dolan et al. 1993	Thailand	Pregnant women	Malaria treatment/prevention	RCT	Effectiveness	Relative risk of at least having one attack of falciparum in pregnant women who presented for an ANC visit and who already had a family size non-impregnated bednet compared to those who received a study non-impregnated bednet in Bono and Maesalit	Risk ratio	58	2.2 (0.9, 5.3)	Refugees	Camp	Doctors, health workers	Clinics, hospitals
Carrara et al. 2017	Thailand	Pregnant women	Micronutrient supplementation	Observational study	Effectiveness	Reduction in SGA in newborns after the increase of uptake of micronutrients and change in food ration in pregnant women	%	0	40.1 (34.7, 45.9)	Refugees	Camp	Health workers	Clinics
Benage et al. 2015	Lebanon	Pregnant women	Screening for referral (other than STIs)	Observational study	Coverage	Proportion of pregnant women receiving all 3 antenatal care interventions (blood pressure measurement, urine and blood sample analysis) out of those who had at least one antenatal care visit	%	348	31.2 (26.33, 36.07)	Refugees	Dispersed	Health workers	Clinics, communal spaces
Mayaud et al. 1997	Tanzania	Pregnant women	STI prevention, treatment and follow-up care	Observational study	Coverage	Proportion of women who had any STD symptom and received treatment out of those enrolled in the study attending ANC clinics	%	63	17 (7.72, 26.28)	Refugees	Camp	Unreported	Clinics
Mayaud et al. 2001	Tanzania	Pregnant women	STI prevention, treatment and follow-up care	Observational study	Effectiveness	Percentage decrease in reproductive tract infections(RTIs) among women attending antenatal care clinics between baseline (1994) and second survey (1996)	%	0	25	Hosts, Refugees	Camp	Trained civilians	Clinics
Augusto et al. 2015	Angola	Pregnant women	STI screening for referral	Observational study	Coverage	Proportion of pregnant women who were tested for HIV at PMTCT sites nationally in 2005	%	12061	1.6 (1.38, 1.82)	Not displaced	N/A	Unreported	Clinics
Augusto et al. 2015	Angola	Pregnant women	STI screening for referral	Observational study	Coverage	Proportion of pregnant women who were tested for HIV at PMTCT sites nationally in 2012	%	314805	33.7 (33.54, 33.87)	Not displaced	N/A	Unreported	Clinics
Bannink-Mbazzi et al. 2013	Uganda	Pregnant women	STI screening for referral	Observational study	Coverage	Proportion of pregnant women who were tested for HIV out of all women newly attending ANC services at the MOH/AVSI health facilities between 2002 and 2011	%	140658	94.4 (86.8, 99)	IDPs, not displaced	Camp	Unreported	Clinics

Author (year)	Country	Target population	Intervention	Report type	Outcome	Description of outcome	Effect measure	Sample size	Estimate (95% CI)	Displace ment status	Setting	Personnel	Site of delivery
Viswanat han et al. 2012	Afghanistan	Women 10-49 years	Training	Observational study	Effective ness	Odds of going to antenatal care during pregnancy in women in villages who had at least one female CHW compared to those that didn't have any CHW, among all surveyed women aged 10-49 years who had delivered a live birth in the 2 years prior to the survey	Odds ratio	3098	2.7 (1.87, 3.92)	Not displaced	N/A	Health workers	Clinics
Benage et al. 2015	Lebanon	Pregnant women	Vaccinations	Observational study	Coverage	Proportion of pregnant women receiving tetanus prophylaxis out of those who had at least one antenatal care visit	%	348	8 (5.15, 10.85)	Refugees	Dispersed	Health workers	Clinics, communal spaces
**Labour and delivery**													
Adam et al. 2015	Sudan	Women 15-49 years	Behavioural education	Observational study	Coverage	Women receiving maternal health education at home among those who gave birth in the last 2 years	%	640	86.7 (84.2, 89.4)	IDPs	Camp	Community health workers	Home
Adam et al. 2015	Sudan	Women 15-49 years	Behavioural education	Observational study	Effective ness	Adjusted odds ratio of delivering at home in women who received maternal health education at home compared to those who didn't	Odds ratio	236	0.6 (0.35, 0.93)	IDPs	Camp	Community health workers	Home
Kruk et al. 2010	Liberia	Pregnant women	EmOC	Observational study	Coverage	Proportion of the surveyed population in Nimba county who could access EmOC services	%	1434	26.8 (24.51, 29.09)	Not displaced	Not reported	Doctors, nurses, health workers	Clinics, hospitals, health posts
Odero et al. 2001	Kenya	Pregnant women	EmOC	Observational study	Coverage	Rate of major obstetric interventions for all indications (maternal and foetal) in all expected births in refugee women who delivered between January 1996 and September 1999	Rate	4280	3.1	Hosts, IDPs	Camp	Nurses, health workers	Hospitals
Odero et al. 2001	Kenya	Pregnant women	EmOC	Observational study	Coverage	Rate of major obstetric interventions for all indications (maternal and foetal) out of all expected births in pregnant women from the host population who delivered between January 1996 and September 1999	Rate	7630	2.1	Hosts, IDPs	Camp	Nurses, health workers	Hospitals

Author (year)	Country	Target population	Intervention	Report type	Outcome	Description of outcome	Effect measure	Sample size	Estimate (95% CI)	Displace ment status	Setting	Personnel	Site of delivery
Orach et al. 2004	Uganda	Pregnant women	EmOC	Observational study	Coverage	Rate of major obstetric interventions for absolute maternal indication in Adjumani district out of all expected births in pregnant host women who stay in rural areas without refugees	%	24670	0.6 (0.54, 0.74)	Hosts, Refugees	Camp	Unreported	Hospitals
Orach et al. 2004	Uganda	Pregnant women	EmOC	Observational study	Coverage	Rate of major obstetric interventions for absolute maternal indication in Adjumani district out of all expected births in pregnant Refugee women	%	4424	1.2 (0.92, 1.59)	Hosts, Refugees	Camp	Unreported	Hospitals
Orach et al. 2004	Uganda	Pregnant women	EmOC	Observational study	Coverage	Number of major obstetric intervention for absolute maternal indication out of all expected births in pregnant refugee women in the West Nile region between 1999–2001	Number	6425	65	Hosts, Refugees	Camp	Unreported	Hospitals
Orach et al. 2004	Uganda	Pregnant women	EmOC	Observational study	Coverage	Number of major obstetric interventions for absolute maternal indication out of all expected births in pregnant hosts living in rural areas with refugees in the West Nile region between 1999–2001	Number	30799	141	Hosts, Refugees	Camp	Unreported	Hospitals
Orach et al. 2004	Uganda	Pregnant women	EmOC	Observational study	Coverage	Number of major obstetric interventions for absolute maternal indication out of all expected births in pregnant hosts living in rural areas without refugees in the West Nile region between 1999–2001	Number	115789	464	Hosts, Refugees	Camp	Unreported	Hospitals
Orach et al. 2004	Uganda	Pregnant women	EmOC	Observational study	Coverage	Number of major obstetric interventions for absolute maternal indication out of all expected births in pregnant hosts living in urban areas in the West Nile region between 1999–2001	Number	28751	296	Hosts, Refugees	Camp	Unreported	Hospitals
Van Damme et al. 1998	Guinea	Pregnant women	EmOC	Observational study	Coverage	Estimated rate ratio of major obstetric interventions in areas with high numbers of refugees overtime between before their arrival (1988-90) and after (1994-96)	Rate ratio	0	4.4 (2.64, 7.15)	Hosts, Refugees	Both settings	Doctors, health workers	Hospitals

Author (year)	Country	Target population	Intervention	Report type	Outcome	Description of outcome	Effect measure	Sample size	Estimate (95% CI)	Displace ment status	Setting	Personnel	Site of delivery
Van Damme et al. 1998	Guinea	Pregnant women	EmOC	Observational study	Coverage	Estimated rate ratio of major obstetric interventions in areas with medium numbers of refugees overtime between before their arrival (1988-90) and after (94-96)	Rate ratio	0	1.7 (1.4, 2.07)	Hosts, Refugees	Both settings	Doctors, health workers	Hospitals
Van Damme et al. 1998	Guinea	Pregnant women	EmOC	Observational study	Coverage	Estimated rate ratio of major obstetric interventions in areas with low numbers of refugees overtime between before their arrival (1988-90) and after (1994-96)	Rate ratio	0	1.9 (0.97, 3.87)	Hosts, Refugees	Both settings	Doctors, health workers	Hospitals
Tatah et al. 2016	Cameroon	Pregnant women	EmOC (Cesarean section)	Observational study	Coverage	The difference in proportion of women having a caesarean delivery in 2011 in the refugee hosting community compared to a control group selected through propensity score matching from the rest of the country.	%	15426	1.2 (1.03, 1.37)	Hosts, Refugees	Not reported	Unreported	Clinics
Deboutte et al. 2013	Democratic Republic of Congo	Pregnant women	EmOC (Cesarean section)	Observational study	Coverage	Proportion of C-sections performed out of the total expected live births over a period of 6 months	%	4925	9.7 (8.87, 10.53)	IDPs, not displaced	Dispersed	Doctors, OB/GYN specialists	Clinics, hospitals
Huster et al. 2014	Lebanon	Pregnant women	EmOC (Cesarean section)	Observational study	Coverage	Proportion of pregnant Syrian refugee women who delivered by C-section out of all Syrian refugee women who delivered through UNHCR-contracted hospitals in Lebanon between January and June 2013	%	6366	35.3 (34.12, 36.47)	Refugees	Not reported	Doctors, skilled birth attendants	Hospitals
Simetka et al. 2002	Sri Lanka	Pregnant women	EmOC (Cesarean section)	Observational study	Coverage	Proportion of women who delivered by caesarean section out of all women at who delivered at Mallavi hospital during November 2000-April 2001	%	685	17.4 (14.56, 20.24)	IDPs	Dispersed	Doctors, nurses, OB/GYN specialists, skilled birth attendants, volunteer attendants	Hospitals
White et al. 2012	Thailand	Pregnant women	EmOC (Cesarean section)	Mixed methods	Coverage	Proportion of live births delivered by caesarean section out of all live births in the study area	%	1404	4.8 (3.68, 5.92)	Refugees	Camp	Skilled birth attendants	Clinics, hospitals

Author (year)	Country	Target population	Intervention	Report type	Outcome	Description of outcome	Effect measure	Sample size	Estimate (95% CI)	Displacement status	Setting	Personnel	Site of delivery
Simetka et al. 2002	Sri Lanka	Pregnant women	Malaria treatment/prevention	Observational study	Coverage	Proportion of women who had received chloroquine tablets for malaria prophylaxis out of all women at who delivered at Mallavi hospital during November 2000-April 2001	%	685	17.4 (14.57, 20.24)	IDPs	Dispersed	Doctors, nurses, OB/GYN, skilled birth attendants, volunteers	Hospitals
Fujiya et al. 2007	Palestine	Pregnant women	Health insurance provision	Observational study	Coverage	Proportion of live births in government hospitals in 2000 before the implementation of the Intifada insurance, out of the total reported number of live births in the Bethlehem district	%	5209	22.9 (21.76, 24.04)	Refugees, not displaced	Camp	Unreported	Unreported
Fujiya et al. 2007	Palestine	Pregnant women	Health insurance provision	Observational study	Coverage	Proportion of live births in government hospitals in 2002 after the implementation of the Intifada insurance, out of the total reported number of live births in the Bethlehem district	%	4654	32 (30.66, 33.34)	Refugees, not displaced	Camp	Unreported	Unreported
Bouchghoul et al. 2015	Jordan	Pregnant women	Safe delivery	Observational study	Coverage	Monthly delivery rate in pregnant Syrian refugee women who who came to the GSF unit for delivery in September 2012	Rate	0	5	Refugees	Camp	Skilled birth attendants, OB/GYN specialists	Clinics
Bouchghoul et al. 2015	Jordan	Pregnant women	Safe delivery	Observational study	Coverage	Monthly delivery rate in pregnant Syrian refugee women who who came to the GSF unit for delivery in February 2013	Rate	0	112	Refugees	Camp	Skilled birth attendants, OB/GYN specialists	Clinics
Carrara et al. 2011	Thailand	Pregnant women	Safe delivery	Observational study	Coverage	Proportion of pregnant women who gave birth at home out of all women surveyed for the 1995-97 survey	%	743	80 (77.12, 82.88)	Refugees	Both settings	Health workers, skilled birth attendants	Clinics
Carrara et al. 2011	Thailand	Pregnant women	Safe delivery	Observational study	Coverage	Proportion of pregnant women who gave birth in SMRU clinics out of women surveyed for the 2003 & 2008 survey	%	1681	70 (67.81, 72.20)	Refugees	Both settings	Health workers, skilled birth attendants	Clinics
Carrara et al. 2017	Thailand	Pregnant women	Safe delivery	Observational study	Coverage	Proportion of women who gave birth with skilled birth attendants	%	0	75	Refugees	Camp	Health workers	Clinics
Deboutte et al. 2013	Democratic Republic of Congo	Pregnant women	Safe delivery	Observational study	Coverage	Proportion of deliveries in government registered health structures over 6 months out of the total number of expected live births	%	4925	84.3 (83.28, 85.32)	IDPs, not displaced	Dispersed	Doctors, OB/GYN specialists	Clinics, hospitals

Author (year)	Country	Target population	Intervention	Report type	Outcome	Description of outcome	Effect measure	Sample size	Estimate (95% CI)	Displace ment status	Setting	Personnel	Site of delivery
Deboutte et al. 2013	Democratic Republic of Congo	Pregnant women	Safe delivery	Observational study	Effective ness	Number of maternal deaths avoided in 2008 due to improved access as part of humanitarian assistance through the NGO hospital performing C-sections	Number	304	228 (178, 282)	IDPs, not displaced	Dispersed	Doctors, OB/GYN specialists	Clinics, hospitals
Von Roenne et al. 2010	Guinea	Pregnant women	Safe delivery	Report	Coverage	Proportion of pregnant refugee women who delivered at a health facility assisted by RHG midwives out of all expected deliveries in the study area in 1999	%	13889	24 (23.29, 24.71)	Hosts, Refugees	Both settings	Skilled birth attendants	Clinics, hospitals
Sami et al. 2017	South Sudan	Pregnant women	Screening for referral (other than STIs)	Observational study	Coverage	The difference between the use of a partograph among women who delivered in a primary health care center compared to those who delivered in a hospital out of all women who arrived for delivery at a study facility during the 9-week enrolment	%	343	59.6 (48.1, 70.2)	IDPs, Refugees	Camp	Skilled birth attendants, traditional birth attendants	Clinics, hospitals
Viswanathan et al. 2012	Afghanistan	Women 10-49 years	Training	Observational study	Effective ness	Odds of skilled birth attendance in women in villages who had at least one female CHW compared to those that didn't have any CHW, among all surveyed women aged 10-49 years who had delivered a live birth in the 2 years prior to the survey	Odds ratio	3118	1.8 (1.18, 2.58)	Not displaced	N/A	Health workers	Clinics
Simetka et al. 2002	Sri Lanka	Pregnant women	Vaccinations	Observational study	Coverage	Proportion of women who had received tetanus toxoid vaccination out of all women at who delivered at Mallavi hospital during November 2000-April 2001	%	685	95.1 (93.48, 96.72)	IDPs	Dispersed	Doctors, nurses, OB/GYN specialists, skilled birth attendants, volunteer attendants	Hospitals
**Postnatal care**													
Purdin et al. 2009	Pakistan	Post-natal mothers	Postnatal care	Observational study	Coverage	Proportion of Afghan refugee women who received postnatal care within 72 hours in 2000 as part of the IRC primary health care program	%	0	27.2	Hosts, Refugees	Camp	Health workers	Clinics

Author (year)	Country	Target population	Intervention	Report type	Outcome	Description of outcome	Effect measure	Sample size	Estimate (95% CI)	Displacement status	Setting	Personnel	Site of delivery
Purdin et al. 2009	Pakistan	Post-natal mothers	Postnatal care	Observational study	Coverage	Proportion of Afghan refugee women who received postnatal care within 72 hours in 2006 as part of the IRC primary health care program	%	0	84.5	Hosts, Refugees	Camp	Health workers	Clinics
von Roenne et al. 2010	Guinea	Post-natal mothers	Postnatal care	Report	Coverage	Proportion of women who delivered with RHG assistance who came back for a post-natal consultation in 1999	%	4413	56 (54.54, 57.46)	Hosts, Refugees	Both settings	Skilled birth attendants	Clinics, hospitals
**General Reproductive Health**													
McPherson et al. 2006	Nepal	Women 15-49 years, pregnant women, postnatal mothers	Behavioural education	Observational study	Coverage	Proportion of women who were exposed to the BPP out of all surveyed women who had an infant of less than one year at the time of survey	%	0	54	Not displaced	N/A	Community health workers, skilled and traditional birth attendants	Hospitals, clinics, health posts, home
McPherson et al. 2006	Nepal	Women 15-49 years, pregnant women, postnatal mothers	Behavioural education	Observational study	Effectiveness	Birth-preparedness index (BPI) at baseline in surveyed women who had an infant of less than one year at the time of survey	%	0	33	Not displaced	N/A	Community health workers, skilled and traditional birth attendants	Hospitals, clinics, health posts, home
McPherson et al. 2006	Nepal	Women 15-49 years, pregnant women, postnatal mothers	Behavioural education	Observational study	Effectiveness	Birth-preparedness index (BPI) at endline in surveyed women who had an infant of less than one year at the time of survey	%	0	54	Not displaced	N/A	Community health workers, skilled and traditional birth attendants	Hospitals, clinics, health posts, home

Munyuzangabo M, *et al. BMJ Global Health* 2021; 5:e003750. doi: 10.1136/bmjgh-2020-003750

BMJ Publishing Group Limited (BMJ) disclaims all liability and responsibility arising from any reliance placed on this supplemental material which has been supplied by the author(s)

Appendix D: Reported coverage and effectiveness of neonatal health interventions

Author (year)	Country	Intervention	Report type	Outcome	Description of outcome	Effect measure	Sample size	Estimate (95%)	Displacement status	Setting	Personnel	Site of delivery
Augusto et al. 2015	Angola	HIV prevention, treatment and follow-up care	Observational study	Coverage	Proportion of HIV exposed infants (HEI) identified through PMTCT services who received ARV prophylaxis nationally in 2012	%	-	13	Not displaced	N/A	Unreported	Clinics
Augusto et al. 2015	Angola	HIV prevention, treatment and follow-up care	Observational study	Coverage	Proportion of HIV exposed infants (HEI) identified through PMTCT services who received ARV prophylaxis nationally in 2005	%	-	2	Not displaced	N/A	Unreported	Clinics
White et al. 2012	Thailand	Breastfeeding initiation	Mixed methods	Coverage	Proportion of in term mother-newborn pairs initiating breastfeeding within the first hour after birth out of all evaluable mother-newborn pairs in the study area in 2010	%	902	91.2 (89.35, 93.05)	Refugees	Camp	Skilled birth attendants	Clinics, hospitals
White et al. 2012	Thailand	Breastfeeding initiation	Mixed methods	Coverage	Proportion of preterm mother-newborn pairs who initiated breastfeeding within the first hour after birth out all evaluable preterm mother-newborn pairs in the study area in 2010	%	80	49 (38.05, 59.95)	Refugees	Camp	Skilled birth attendants	Clinics, hospitals
White et al. 2012	Thailand	Breastfeeding initiation	Mixed methods	Effectiveness	Proportion of preterm mother-newborn pairs who were exclusively breastfeeding at time of discharge out all evaluable preterm mother-newborn pairs in the study area in 2010	%	80	98.8 (96.41, 101.2)	Refugees	Camp	Skilled birth attendants	Clinics, hospitals
Sami et al. 2017	South Sudan	Essential newborn care (KMC)	Observational study	Coverage	Proportion of mothers who placed their newborn skin-to-skin within the first hour of birth out of all women who arrived at the study hospital for delivery during the 9-week enrolment	%	153	88.9 (83.92, 93.88)	IDPs, Refugees	Camp	Skilled birth attendants, traditional birth attendants	Clinics, hospitals
Sami et al. 2017	South Sudan	Essential newborn care (KMC)	Observational study	Coverage	Proportion of mothers who placed their newborn skin-to-skin within the first hour of birth out of all women who arrived at the study primary health care centers for delivery during the 9-week enrolment	%	190	49.7 (42.59, 56.81)	IDPs, Refugees	Camp	Skilled birth attendants, traditional birth attendants	Clinics, hospitals

Author (year)	Country	Intervention	Report type	Outcome	Description of outcome	Effect measure	Sample size	Estimate (95%)	Displacement status	Setting	Personnel	Site of delivery
Turner et al. 2013 (PLoS ONE)	Thailand	Training	Observational study	Effectiveness	Proportion of infants who died following admission to the Special Care Baby Unit in 2008	%	181	12.2 (7.43, 16.97)	Refugees	Camp	Paediatrician, doctor, nurses, medics, skilled birth attendants	Clinics
Turner et al. 2013 (PLoS ONE)	Thailand	Training	Observational study	Effectiveness	Proportion of infants who died following admission to the Special Care Baby Unit in 2011	%	259	3.1 (0.99, 5.21)	Refugees	Camp	Paediatrician, doctor, nurses, medics, skilled birth attendants	Clinics
